# Videos of common activities reveal movement patterns associated with biopsychosocial low back function in a worldwide cohort

**DOI:** 10.1177/20552076261477543

**Published:** 2026-08-08

**Authors:** Carl J. Alano, Michael W. R. Holmes, Shawn M. Beaudette

**Affiliations:** 1 Department of Kinesiology, 7497 Brock University, St. Catherines, ON, Canada

**Keywords:** low back pain, biopsychosocial model, human pose estimation, motor phenotypes, telehealth

## Abstract

**Objective:**

Low back pain (LBP) is frequently classified as non-specific, which reduces the capacity of the healthcare system to offer targeted rehabilitation. Previous work has suggested a link between motor control and low back dysfunction. Identifying motor control phenotypes indicative of dysfunction often requires complex and costly laboratory equipment. Recent advancements in computer vision and the widespread availability of smartphones have made human motion capture more accessible. This study aimed to examine whether outcomes derived from consumer-grade video and open-source pose estimation tools are associated with motor control patterns linked to self-reported low back dysfunction.

**Methods:**

A self-guided online questionnaire was employed to gather data from 448 participants, worldwide. Participants completed validated questionnaires and video-recorded themselves performing four functional movements. Pose-derived kinematic features were extracted and reduced using principal component analysis (PCA), followed by a stepwise linear regression modelling to examine associations between movement features and individual participant reported outcome measures as well as a composite index of low back function. Participants were split into low and high function groups.

**Results:**

PCA-derived features of movement were significantly associated with measures of disability, kinesiophobia, pain catastrophizing, and physical activity. Trunk flexion demonstrated the strongest association with the composite index (R^2^ = 0.71). The results between low vs high function participants depict biomechanically relevant differences (i.e. reduced movement speed and range of motion) typically found in the low back pain (LBP) population.

**Conclusion:**

Results highlight the feasibility of using consumer-grade video and open-source pose estimation tools for large-scale biomechanical data collections, to enhance our understanding of LBP. Although strong associations were observed between video-derived movement features and self-reported dysfunction, prospective validation and external testing may be required before clinical screening performance can be established. With appropriate validation, this approach has the potential to support the development of a scalable and accessible digital movement assessment tool for low back dysfunction.

## 1. Introduction

Low back pain (LBP) is the leading cause of disability worldwide, affecting >500 million annually.^1^ Up to 90% of LBP cases are non-specific,^2^ suggesting pain cannot be attributed to any specific pathology.^3^ This complexity of LBP is best understood through the biopsychosocial (BPS) model, which considers the interaction of physical, psychological, and social factors in the development and management of pain.^4^ Despite the widespread acceptance of the BPS model, clinical management often address symptoms while overlooking the underlying causes of low back disorder (LBD).^5^ As a result, LBDs that may otherwise be treatable persist into chronic and/or recurrent cases, further increasing the already large socioeconomic burden.^6^

Increasing evidence suggests that motor dysfunction, characterized by altered movement and coordination patterns, contributes to the persistence of pain in individuals with LBP.^7^ Importantly, altered motor strategies in those with LBP may reflect adaptive or protective responses to pain or perceived threat which may also have secondary consequences (e.g. altered loading and reduced variability) that could contribute to ongoing symptoms in some individuals.^8^ Recognizing this apparent heterogeneity in motor control phenotypes through the intersection of biological, psychological and social considerations could allow for more personalized and effective treatment strategies.^9^ However, current assessments of spine movement quality remain subjective and unreliable among healthcare professionals, limiting their clinical utility.^10^ Therefore, objective and scalable assessment tools for motor control are urgently needed, including an enhanced understanding of how motor adaptations related to common psychosocial factors associated with LBP.^11^ Technologies such as 3D motion capture have enabled precise analysis of human movement but are often impractical for widespread clinical use due to cost and complexity. Recent advancements in computer vision, specifically deep convolutional neural networks, and widespread smartphone availability have opened new avenues for accessible, affordable, and scalable video-based motion tracking (human pose estimation). These technologies enable efficient tracking of movements, facilitating large-scale data collection and analysis.^12^ Moreover, video-based assessments can be administered remotely through telehealth or e-health platforms, offering significant opportunities to improve care delivery and expand access for underserved populations.

With the improved availability of human movement data, the next challenge comes with the management and reduction of such data. Principal component analysis (PCA) has been widely used to objectively reduce complex kinematic data into clinically meaningful modes of variation across diverse tasks such as lifting,^13^ gait,^14^ and athletics.^15,16^ PCA-derived motor phenotypes have successfully differentiated performance and functional outcomes in various contexts, including athletic performance, gender, and pathological movement strategies.^17,18^ However, there is currently a critical gap in understanding how PCA-based approaches to video-derived motion data specifically correlate with participant-reported outcome measures (PROMs) relevant to LBP, such as disability, pain catastrophizing, kinesiophobia, and physical activity. Given that low back dysfunction is multidimensional within the BPS model, it could be possible to represent PROMs using an exploratory composite index intended to simplify the data without sacrificing underlying information. This approach has been utilized previously for composite patient reported-outcome construction.^19^

Therefore, this study aimed to leverage open-source human pose estimation software (MediaPipe™, Google) to objectively classify motor phenotypes derived from smartphone videos and evaluate their association with participant-reported low back function outcomes. It was hypothesized that PCA-derived motor phenotypes characterized by slower movement speed and reduced range of motion would be associated with poorer patient-reported metrics of low back function, specifically higher disability, greater pain catastrophizing, higher kinesiophobia, and lower physical activity. This work would constitute and important first step in the development of a scalable and objective framework for identifying movement patterns relevant to low back function and managing of low back dysfunction.

## 2. Methodology

### 2.1. Participants and procedures

#### 2.1.1. Participants

This cross-sectional observational study was conducted remotely, with participants recruited through various channels, including word of mouth, posters, social media, and an online recruitment platform (Prolific^TM^, UK). All study procedures were completed in participants’ environments and were submitted online on May 26, 2023. For Prolific^TM^ recruitment, participant was restricted to a single submission per unique Prolific ID. Submissions were reviewed prior to compensation to confirm that a valid video file was provided. All recordings were subsequently manually screened for protocol adherence and video quality across all tasks, with exclusions applied as described in Section 2.3 and detailed in the supplementary material. To qualify for the study, participants had to meet the following criteria: (1) be aged 18-85 years, (2) not have any diagnosed musculoskeletal disorders affecting movement or balance, (3) not have experienced a lower limb injury requiring medical intervention in the past three months, and (4) be able to self-record while performing specified movements. All participants provided informed consent prior to participation in accordance with the Tri-council policy statement (TCPS) regarding the ethical conduct or research involving humans. A formal *a priori* sample size calculation was not performed because this study was exploratory in nature and involved high-dimensional whole body kinematic data, with movement-specific exclusions after video quality screening and preprocessing. Instead, sampling was terminated at a target n = 450, which was selected to meet or exceed sample sizes reported in comparable prior work using a similar remote data collection approach (e.g., n = 405), while also remaining substantially larger than typical biomechanical study samples.^12^

#### 2.1.2. Inclusion and exclusion criteria

Participants who did not meet the eligibility criteria or failed to provide complete data were excluded from the final analysis. Additional exclusions were applied when procedural or recording errors prevented reliable pose extraction or task interpretation. Common reasons for exclusions included improper camera angle or distance, incomplete movement execution, camera movement during recording, lighting problems, clothing related landmark occlusion, as described further in the Data Processing and Analysis section. The final sample included participants who successfully completed all the tasks and passed the necessary data quality checks. Detailed task-specific exclusion flowcharts are provided in the Supplementary Material.

#### 2.1.3. Procedures

**Figure 1.**
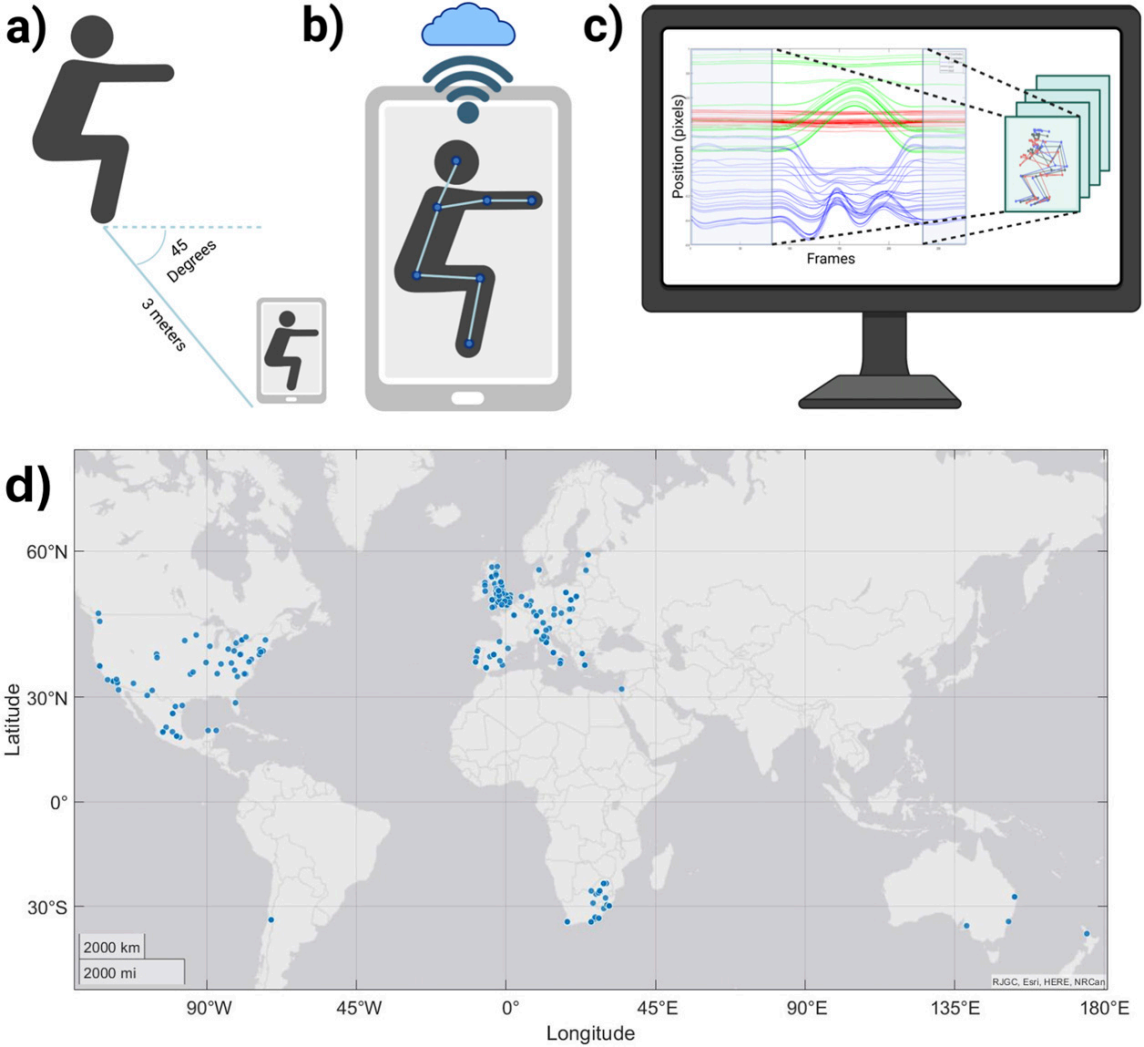
Data acquisition process. (a) Participants captured smartphone video for all tasks at a distance from 3m away at the approximate 9 o’clock position. (b) videos from all data were uploaded to a cloud storage server for subsequent pose estimation analysis. (c) 3D pose-estimation data were downloaded, visually inspected, and pre-processed to facilitate the development of a database for each movement subtype. (d) The decentralized nature of the study facilitated the acquisition of data with representation from North America, South America, Europe, South Africa, and Australia.

Following informed consent, participants completed a screening questionnaire about their musculoskeletal health, including current pain in the lower back and lower extremities. Participants then recorded themselves performing one repetition of four activities of daily living (ADLs): squat, small-object pickup, trunk flexion, and sit-to-stand/stand-to-sit (e.g., Figure 1). After these tasks, participants completed several validated self-report questionnaires capturing low-back disability, including the Oswestry Disability Index (ODI),^20,21^ Quebec Back Pain Disability Scale (QBPDS),^22,23^ Tampa Scale for Kinesiophobia (TSK),^24,25^ Pain Catastrophizing Scale (PCS),^26^ and International Physical Activity Questionnaire (IPAQ),^27^ which were used to assess low-back disability, kinesiophobia, pain catastrophizing, and physical activity levels.

#### 2.1.4. Activities of daily living (ADLs)

The ADLs assessed in this study were selected to capture common movements that may be impacted by lower back pain. To evaluate these tasks in a standardized and repeatable manner, participants were instructed to perform each movement, as detailed below:

Squat: Participants were instructed to stand with their legs shoulder width apart and complete a single body weight squatting motion. To accomplish this, they were asked to descend as far as they felt comfortable, at their own self-selected pace, before returning to their original standing position (Supplementary Figure 1).

Small-object (i.e., pen) pickup: Participants were instructed to place the pen ∼30cm away on the floor directly in front of their feet. They were then asked to bend forward to grasp the object with their right hand and lift the object to waist height and return it to the original position at their own self-selected pace (Supplementary Figure 3).

Trunk flexion: Participants were instructed to stand with their legs shoulder width apart. While in this position, they were instructed to round their spine as much as they felt comfortable, before returning to their original position (Supplementary Figure 6).

Sit-to-stand/stand-to-sit: Participants were instructed to start in a seated position with their hands across their chest. While in this position, they were asked to stand up and sit back down five consecutive times (Supplementary Figure 9).

### 2.2. Survey measures

#### 2.2.1. Participant demographics

Participants provided demographic information, including age, sex, height, LBP status, and mass. Body mass index (BMI) was calculated from self-reported data.

#### 2.2.2. Low-back related disability

The Oswestry Disability Index (ODI)^20,21^ and the Quebec Back Pain Disability Scale (QBPDS)^22,23^ were used to assess the impact of lower back pain on daily function. ODI scores provided a measure of disability, while QBPDS scores assessed functional limitations related to pain. Higher scores on both scales indicate greater disability.

#### 2.2.3. Kinesiophobia

Kinesiophobia, or fear of movement due to pain, was measured using the Tampa Scale for Kinesiophobia (TSK).^24,25^ Participants rated their fear of physical activity on a scale from low to high.

#### 2.2.4. Pain catastrophizing

The Pain Catastrophizing Scale (PCS)^26^ was used to assess participants’ emotional responses to pain, including feelings of helplessness, rumination, and magnification.

#### 2.2.5. Physical activity level

The International Physical Activity Questionnaire (IPAQ)^27^ was used to evaluate participants’ weekly physical activity. Activity levels were quantified in MET-minutes per week.

#### 2.2.6. Biopsychosocial composite index model

For each questionnaire category (ODI, QBPDS, TSK, PCS, and IPAQ), the mean and standard deviation were calculated. Individual questionnaire scores were then standardized into z-scores to allow comparison across different scales. To calculate a Composite Index (CI) representative of overall low back function, the z-scores from each questionnaire were combined following methods outlined by Chan et al. (2020).^28^ Specifically, because ODI and QBPDS measure similar aspects of low back pain-related disability, each was assigned a weight of 0.5 to represent a single disability domain and minimize redundancy between these overlapping measures, while TSK, PCS, and IPAQ were assigned a weight of 1. The weighted z-scores were summed and then divided by 4, yielding the final composite index score:

$$Equation\;1:CI=\frac{\frac{1}{2}{\;z}_{ODI}+\frac{1}{2}{\;z}_{QBPDS}+z_{TSK}+z_{PCS}+z_{IPAQ}}{4}$$

### 2.3. Video analysis

#### 2.3.1. Automated pose estimation

Participant videos were analyzed using MediaPipe^TM^, an open-source 3D pose estimation tool. The tool tracks 33 anatomical landmarks and outputs 3D coordinates, which were stored in .csv format for subsequent processing in MATLAB (v R2024b, The MathWorks Inc., Natick, MA, USA).

#### 2.3.2. Pre-processing

The videos were manually screened for procedural errors, such as incorrect recording angles or incomplete movements. Flowcharts detailing these errors and the exclusion criteria are provided in supplementary figures (Supplementary Figures 2,4,7,10,13). Manual adjustments were made to ensure consistency, including cropping, and/or mirroring videos where necessary for unilateral movements to ensure spatiotemporal alignment across all videos.

#### 2.3.3. Data reduction and parameter extraction

Following event detection and the manual removal of erroneous 3D pose data, all coordinate data were filtered using a bi-directional, zero-lag, recursive Butterworth filter with an effective low pass cut-off frequency of 6 Hz. Once cropped, all pose data were time-normalized to 101 frames (i.e., 0-100% movement) via the interpolation and subsequent re-sampling of 101 evenly spaced points along a piecewise cubic Hermite interpolating polynomial (PCHIP) for all 3D points within the point cloud. This step preserves spatiotemporal differences in coordination for each movement, but represents all movements of differing pace (i.e., duration) with the same number of frames to facilitate further analyses. Importantly, elapsed time data were also retained as a time-normalized vector in all subsequent analyses to ensure that any variability related to movement timing was retained.

Once time-normalized, all data were de-biased and amplitude normalized such that any variation in the location (i.e., within the camera frame) and size (i.e., height and width of the participant, and approximate location with respect to the camera due to parallax errors) of each participant were removed from any subsequent analyses. To accomplish this, every point cloud was represented relative to the middle 3D point between each of the heel markers by subtracting this point from every other point within the 3D point cloud. In addition, all 3D coordinate data were normalized to the x, y, and z component ranges, such that all 3D data, across all participants of varying heights, widths, and camera locations were represented from 0-1 along each component axis.

Due to variations in the orientation of each participant relative to the recording device, all 3D point clouds required representation within the same global coordinate frame to facilitate further comparison. To accomplish this a local frame was generated to represent the orientation of each individual within the first frame, and all 3D point cloud data were rotated about this local reference frame to ensure consistent alignment of every individual within the database. Specifically, a medial-lateral reference axis was generated by subtracting the 3D position of the right shoulder marker from that of the left. Similarly, a superior-inferior reference axis was generated by subtracting the mean 3D position between the heel markers from the mean 3D position between the hip markers. Each vector was divided by its length to generate unit vectors. The cross product of these two-unit vectors was used to generate a right-handed orthonormal local coordinate system whereby the positive x-axis is oriented posteriorly, the positive y-axis is oriented to the right, and the positive z-axis is oriented superiorly. Remaining point cloud data were rotated to align with this body-referenced local coordinate system to ensure that all data, for all participants, were expressed in comparable reference frames.

Following the pre-processing steps noted above, all 3D point cloud data across all 101 data points were stored in a single data matrix with the following format:

Equation 2:

$$\left[\;\begin{array}{l} t_{1-101}^{1}\\ \vdots \\ t_{1-101}^{n} \end{array}\;\begin{array}{lll} x_{1-101}^{1} & y_{1-101}^{1} & z_{1-101\;}^{1}\\ \vdots & \vdots & \vdots \\ x_{1-101}^{n} & y_{1-101}^{n} & z_{1-101}^{n} \end{array}\;\begin{array}{lll} {vx}_{1-101}^{1} & vy_{1-101}^{1} & vz_{1-101\;}^{1}\\ \vdots & \vdots & \vdots \\ {vx}_{1-101}^{n} & vy_{1-101}^{n} & vz_{1-101}^{n} \end{array}\;\begin{array}{lll} {ax}_{1-101}^{1} & ay_{1-101}^{1} & az_{1-101\;}^{1}\\ \vdots & \vdots & \vdots \\ {ax}_{1-101}^{n} & ay_{1-101}^{n} & az_{1-101}^{n} \end{array}\;\right]$$

Such that $t_{1-101}^{n}$ represents the elapsed time for a single movement trial (in seconds), $x_{1-101}^{n}$ , $y_{1-101}^{n}$ , and $z_{1-101}^{n}$ represents all pre-processed xyz-coordinates, ${vx}_{1-101}^{n}$ , ${vy}_{1-101}^{n}$ , and $vz_{1-101}^{n}$ represents all pre-processed first time derivatives (i.e.,velocity) xyz-coordinates, ${ax}_{1-101}^{n}$ , $ay_{1-101}^{n}$ , and $az_{1-101}^{n}$ represents all preprocessed second time derivatives (i.e., acceleration) xyz-coordinates from all 33 markers for a single movement trial and each row corresponds to a different person included within the dataset.

### 2.4. Statistical analysis

#### 2.4.1. Descriptive statistics

**Figure 2.**
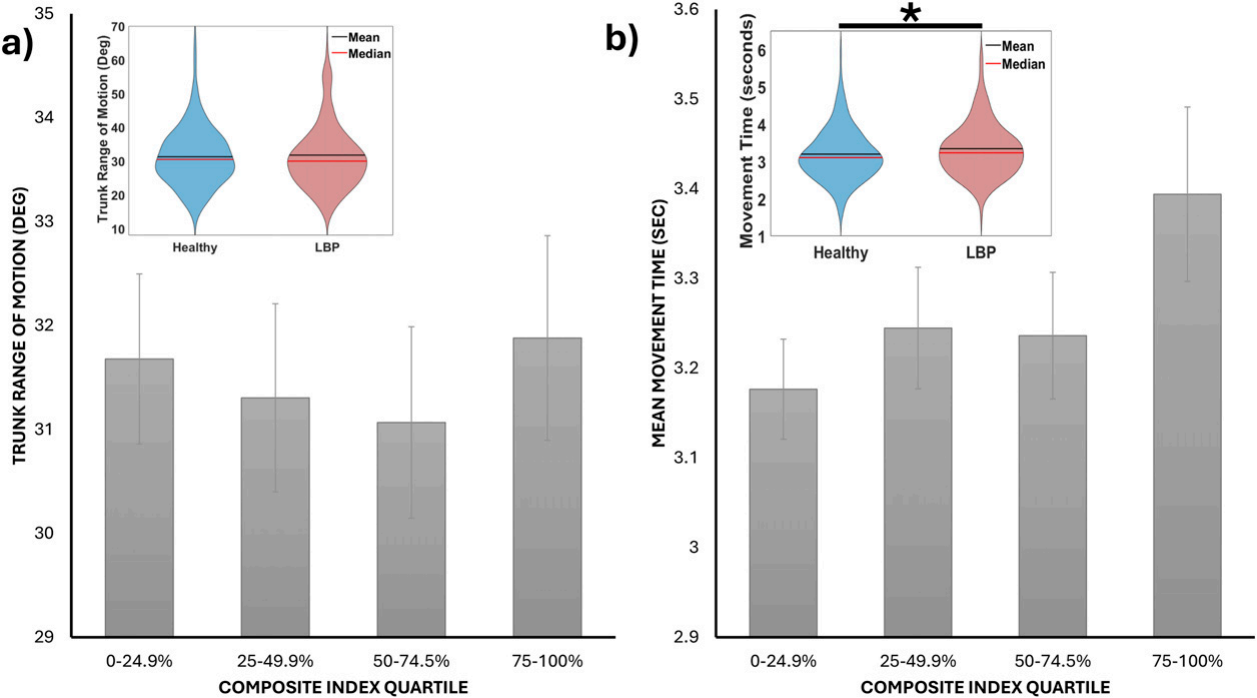
Comparison of trunk range of motion (ROM) and movement time across composite index quartiles. (a) The bar chart shows the mean trunk ROM, which remains relatively stable across quartiles, with a slight variability. The inset violin plot illustrates the distribution of trunk ROM between healthy and LBP participants, with the mean and median marked. (b) The bar chart shows the mean movement time, indicating an increase from Q1 to Q4. The inset violin plot shows the distribution of movement time between healthy and LBP participants, with a statistically significant difference between groups (p < 0.05).

Following data processing, descriptive statistics were calculated for mean movement time and mean trunk inclination range of motion (ROM) across groups and composite index quartiles, using data from all movements. Mean movement time and mean trunk inclination ROM were analyzed to understand variations across healthy individuals and those with LBP, as well as differences across composite index quartiles (Q1–Q4). These descriptive statistics are visualized in Figure 2, which illustrates both the distribution of ROM and movement time across quartiles and between health status groups.

#### 2.4.2. Associations

Following pre-processing steps, the video-based kinematic database (Equation 2) was reduced using PCA. PCA was applied to the preprocessed kinematic database after filtering, time normalization, amplitude normalization, and reference-frame alignment. These pre-processing steps reduced variation related to participant size, location within the frame, and body orientation prior to PCA. For the primary association models, PC scores accounting for up to 99% of the variance were retained for each movement. To assess the influence of PCA dimensionality reduction on model complexity and task-level associations, a secondary analysis was also performed using a more conservative 95% PCA retention threshold. Stepwise linear multi-regression was then conducted five times, once for each movement (squat, small-object pickup, trunk flexion, sit to stand, and stand to sit), as an exploratory association analysis to identify PCA-derived features related to individual PROMs or Composite Index representations of overall function. In this analysis, age, BMI, and individual PC scores from each movement were used as dependent variables, while the composite index, or individual PROMs served as the independent variables, with significant results interpreted at an alpha level of 0.05. Due to variations in numbers of retained videos, each of the five movement databases had different sample sizes, as some videos submitted by participants did not meet data quality standards. PC scores with significant effects were further examined using a single^29^ and aggregate^15^ component reconstruction framework to visually interpret movement phenotypes.

Aggregate reconstructions representing high, mean, and low function levels were created using predicted PC scores derived from the stepwise linear regression model. Specifically, for each PC retained by the regression model, its slope with respect to the composite index was identified as either positive or negative. PCs positively correlated with CI were reconstructed at the 95^th^ percentile of the CI distribution, while PCs negatively correlated were reconstructed at the 5^th^ percentile. Mean function reconstructions used predicted PC scores corresponding to the mean CI values. These percentile-based reconstructions enabled visualization of movement patterns associated with varying levels of function.

## 3. Results

### 3.1. Sample distribution and demographics

The final dataset comprised 448 participants: 224 male, 220 female, and 4 identifying as other (Table 1). Based on the screening questionnaire, 206 participants reported current LBP, and 242 participants reported no current LBP. This balanced distribution enabled a broad characterization of movement patterns across individuals with varying anthropometric profiles, facilitating a more representative sample of the broader population.

**Table 1.** Characteristics of the study participants represented by the Mean (± Standard Deviation).

Demographic	Male	Female	Other
N	224	220	4
Age (years)	33.14 ± 10.79	31.33 ± 9.63	28.25 ± 3.11
Mass (kg)	79.71 ± 18.31	71.82 ± 19.23	63.2 ± 13.82
Height (m)	1.76 ± 0.10	1.62 ± 0.14	1.54 ± 0.03

Prior to aggregation, the PROMs demonstrated moderate to strong intercorrelations among ODI, QBPDS, PCS, and TSK (Spearman p = 0.366-0.766), with the strongest association observed between ODI and QBPDS (p = 0.766). IPAQ showed weak inverse correlations with the other PROMs (p = -0.128 to -0.056), with only the ODI-IPAQ association reaching statistical significance. Multicollinearity diagnostics did not indicate severe redundancy among our selected measures, with VIF values ranging from 1.022 to 3.508 and tolerance ranging from 0.285 to 0.979 (Supplementary Table 1).

### 3.2. Squat

**Figure 3.**
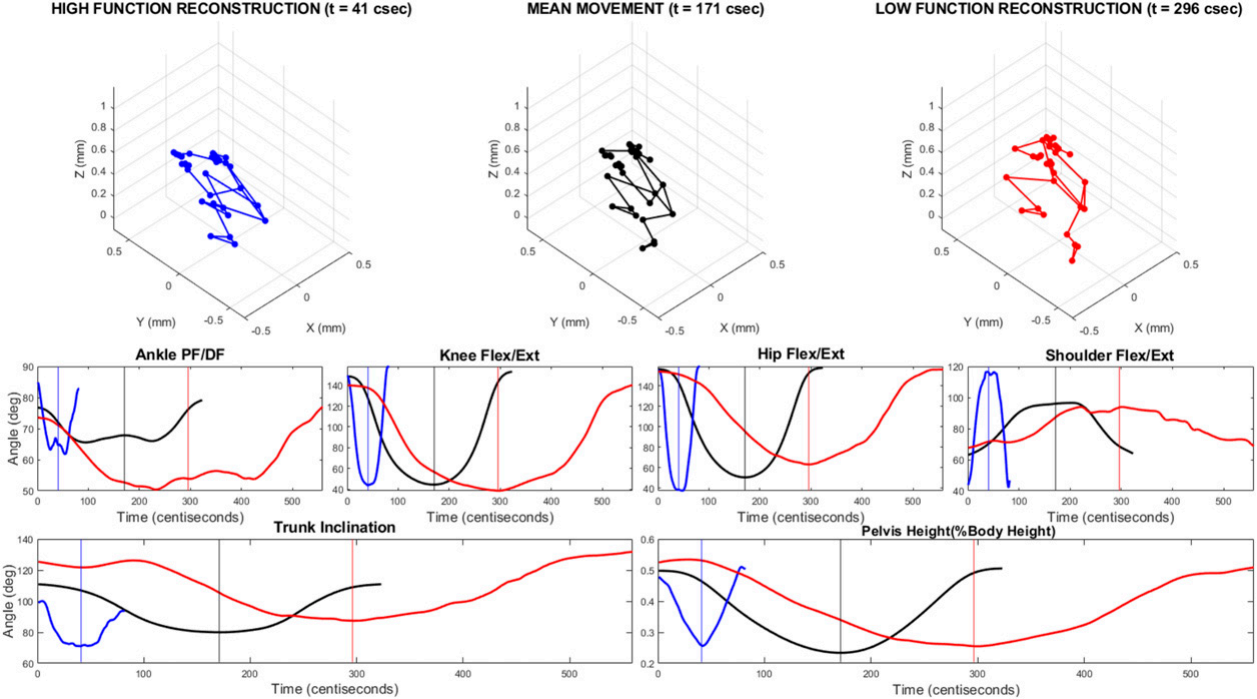
Aggregate reconstruction of squat movement at low, mean, and high composite index levels using stepwise linear regression. Movement reconstructions were generated using predicted PCA scores from a stepwise linear regression model, with the composite index as the predictor. The reconstructions represent low (5th percentile, red), mean (black), and high (95th percentile, blue) CI values. The top row displays a static 3D pose at the moment of the lowest point of pelvis height. The bottom row represents kinematic plots showing angular changes over time for various joints and body parts for the entire movement. The vertical lines represent the lowest point of pelvis height during the movement.

A total of 331 videos were retained for the squat task after removing submissions with procedural or recording errors (Supplementary Figure 2). Following PCA, 205 components were retained, explaining 99.01% of the variance. Aggregate reconstruction from PCs included in stepwise linear regression model revealed functional differences in timing and ROM. High-function individuals reached their lowest pelvis height in 0.41 seconds, while low-function individuals reached the same point at 2.96 seconds, indicating delayed and restricted movement. High-function participants also demonstrated greater hip flexion and trunk inclination compared to the low-function group (Figure 3). Stepwise regression analysis revealed significant associations between PCA-derived kinematics and all PROMs. The composite index stepwise linear regression model retained 41 PCs (R^2^ = 0.42, RMSE = 0.53). Among individual PROMs, the QBPDS presented with the strongest relationship (R^2^ = 0.45), while IPAQ exhibited the weakest (R^2^ = 0.38) (Supplementary Tables 2 and 3).

### 3.3. Small object pickup

A total of 329 videos were retained for this task after exclusion of poor-quality recordings due to framing, lighting, or movement errors (Supplementary Figure 4). Following PCA, 216 components were retained, explaining 99.02% of the variance. Aggregate reconstruction of those PCs included in the stepwise linear regression model revealed functional differences in joint ROM, movement timing, and trunk inclination. High-function individuals reached peak trunk flexion earlier (0.86 secs vs. 4 secs) and demonstrated greater, faster motion at the knee and hip. In contrast, low-function individuals exhibited reduced ROM and slower, more gradual movement patterns (Supplementary Figure 5). Stepwise regression revealed strong associations between motor phenotypes and PROMs. The composite index stepwise linear regression model retained 52 PCs and age as predictors (R^2^ = 0.54, RMSE = 0.48). Among PROMs, the strongest association was with the TSK (R^2^ = 0.56) and the weakest with IPAQ (R^2^ = 0.35) (Supplementary Tables 4 and 5).

### 3.4. Trunk flexion

A total of 324 videos were retained for the trunk flexion task following exclusions related to recording errors (Supplementary Figure 7). Following PCA, 252 components were retained, explaining 99.02% of the variance. Aggregate reconstruction from PCs included in stepwise linear regression model revealed notable functional differences across participants (Supplementary Figure 8). Low-function individuals reached their peak trunk inclination earliest (3.41 secs), while high-function individuals reached this point comparatively later (5.07 secs). The low-function group exhibited increased trunk inclination but limited ROM, suggesting altered strategy and reduced control. In contrast, high-function participants showed greater and more coordinated trunk and lower body motion. Stepwise linear regression models showed strong associations between movement characteristics (trunk flexion) and all PROMs including ODI, QBPDS, PCS, TSK and IPAQ. The composite index stepwise linear regression model showed the strongest in-sample association, with R^2^ = 0.71 and RMSE = 0.41, using 86 PCs and age as predictors. Among individual PROMs, the ODI showed the strongest association (R^2^ = 0.70), while IPAQ again showed the weakest (R^2^ = 0.36) (Supplementary Tables 6 and 7).

### 3.5. Sit-to-stand

A total of 262 videos were retained for the sit-to-stand task after excluding recordings with procedural issues such as incorrect angles or poor visibility or chair-related limb occlusion (Supplementary Figure 10). Following PCA 171 components were retained, accounting for 99.02% of the total variance. Aggregate reconstruction from PCs included in stepwise linear regression model revealed clear functional stratification based on timing and joint behavior (Supplementary Figure 11). High-function participants reached peak trunk inclination at 0.82 seconds, compared to 2.50 seconds in the low-function group. While joint movement patterns (knee, hip, trunk, pelvis) appeared similar in form, the magnitude and speed of execution varied notably across groups. Stepwise linear regression analysis revealed significant associations between movement characteristics and all PROMs. The composite index stepwise linear regression model retained 32 PCs and BMI (R^2^ = 0.44, RMSE = 0.52). The strongest association was with the TSK (R^2^ = 0.52), followed closely by QBPDS, while IPAQ again showed the weakest relationship (R^2^ = 0.41) (Supplementary Tables 8 and 9).

### 3.6. Stand-to-sit

A total of 239 videos were retained for the stand-to-sit task following the exclusion of submissions with significant procedural errors (Supplementary Figure 13). Following PCA 162 components were retained, explaining 99.01% of the variance. Functional differences were observed across participant groups, particularly in timing and movement amplitude (Supplementary Figure 12). High-function participants reached peak trunk inclination at 1.24 seconds, while low-function participants reached it at 2.0 seconds, indicating delayed and less efficient movement. While joint waveforms were qualitatively similar across groups, low-function individuals demonstrated slower execution and lower amplitude in key joints such as the knee, hip, and trunk. Stepwise linear regression models showed significant associations between movement characteristics and all PROMs. The composite index stepwise linear regression model included 28 PCs along with BMI and age (R^2^ = 0.41, RMSE = 0.53). The strongest individual association was found with the QBPDS (R^2^ = 0.50), while the weakest was with IPAQ (R^2^ = 0.29) (Supplementary Tables 10 and 11).

### 3.7. Composite index model comparison across movements

When examining the movement-specific composite index for each task, certain movements have stronger associations with low back function than others (Table 2). For the Small Object Pick-up, age along with 52 PCs was a significant predictor, explaining 25.52% of the variance with an R^2^ of 0.54 and a root mean square error (RMSE) of 0.48. In the Squat task, the retained predictors accounted for 13.30% of the variance with an R^2^ of 0.42 and an RMSE of 0.53. Trunk Flexion performance was also influenced by age and 86 PCs, which explained 34.79% of the variance (highest among the tasks), achieved an R^2^ of 0.71, and the RMSE for this movement was 0.41. The Sit-to-Stand task demonstrated that BMI and 32 PCs are significant predictors, explaining 18.68% of the variance with an R^2^ of 0.44 and an RMSE of 0.52. Finally, the Stand-to-Sit movement was predicted by age, BMI and 28 PCs, accounting for 10.98% of the variance. This model had an R^2^ of 0.41 and an RMSE of 0.53. Based on these values, the strongest-weakest relation and cumulative explained variance (largest-smallest) between movement composite index spans the following order: Trunk Flexion, Small Object Pick-Up, Sit-to-Stand, Squat, and Stand-to-Sit. Detailed task-specific supplementary outputs are provided in Supplementary Tables 2-11, including in-sample model fit statistics and retained predictor coefficients for each movement.

**Table 2.** Results of Statistical Analysis for Composite Index Across All Movements Using a 99% PCA Retention Threshold.

​	Movement specific composite index				
	Squat	Small-object pick-up	Trunk flexion	Sit-to-stand	Stand-to-sit
Sample Size	331	329	324	262	239
Retained Predictors	41	52 + Age	86 + Age	32 + BMI	28 + Age + BMI
Cumulative Explained Variance	13.30	25.52	34.79	18.68	10.99
R^2^	0.42	0.54	0.71	0.44	0.41
RMSE	0.53	0.48	0.41	0.52	0.53
F Stat	5.03	6.03	6.76	5.51	4.86
p-value	<0.001	<0.001	<0.001	<0.001	<0.001
Kolmogorov-Smirnov Statistic	0.18	0.22	0.25	0.19	0.18

Cumulative explained variance refers to the proportion of variance in the original movement dataset captured by the retained principal components, whereas R^2^ reflects the proportion of variance in the composite index explained by the regression model.

A secondary model comparison was also performed using a more conservative 95% PCA retention threshold (Table 3). Across all movements, reducing the PCA threshold decreased the number of retained predictors and lowered the magnitude of in-sample model fit compared with the 99% PCA model. However, trunk flexion remained the strongest movement-specific model, while sit-to-stand remained the weakest. The ranked order of association strength changed slightly under the 95% threshold with sit-to-stand demonstrating the second strongest association, followed by small-object pick up, squat and stand-to-sit.

**Table 3.** Results of Statistical Analysis for Composite Index Across All Movements Using a 95% PCA Retention Threshold.

​	Movement specific composite index				
	Squat	Small-object pick-up	Trunk flexion	Sit-to-stand	Stand-to-sit
Sample Size	331	329	324	262	239
Retained Predictors	17	18 + Age	46 + Age	15 + Age + BMI	9 + BMI
Cumulative Explained Variance	11.44	21.89	21.69	15.12	7.85
R^2^	0.22	0.24	0.50	0.29	0.16
RMSE	0.59	0.58	0.50	0.57	0.60
F Stat	5.06	5.09	5.77	5.76	4.34
p-value	<0.001	<0.001	<0.001	<0.001	<0.001
Kolmogorov-Smirnov Statistic	0.66	0.50	0.36	0.52	0.74

Cumulative explained variance refers to the proportion of variance in the original movement dataset captured by the retained principal components, whereas R^2^ reflects the proportion of variance in the composite index explained by the regression model.

## 4. Discussion

The current study aimed to bridge the gap between accessible, scalable technologies for human movement analysis and the clinical assessment of motor control associated with LBP. Specifically, we examined the association between objectively derived motor patterns captured via consumer-grade smartphone video and PROMs of low back dysfunction. Results support the hypothesis that motor strategies are significantly associated with key biopsychosocial outcomes related to LBP, such as disability, kinesiophobia, pain catastrophizing, and physical activity. Following the analysis of descriptive statistics, and data-driven reconstructions of movement notable physical changes in movement include changes in movement velocity, ROM, and motor coordination (i.e., sequencing); however, many of the observed changes were task dependent. Furthermore, different tasks demonstrated stronger associations with PROMS, and composite indices of low back function, suggesting movements include spine flexion-extension may be best to discriminate high vs. low function groups.

One of the primary challenges in clinical movement assessment remains subjectivity and low inter-rater reliability, which limits the consistency and accuracy across providers.^10^ The current study aims to address this challenge by employing accessible technology—consumer-grade smartphone video analyzed via open-source pose estimation tools—to quantify whole-body motor patterns. Notably, our study achieved a scale extremely uncommon in traditional biomechanical research, collecting data from 448 participants across 36 countries, resulting in 1,517 usable videos, approximately 35 times larger than typical biomechanical sample sizes. This global approach enhances the generalizability of our findings and captures diverse motor strategies across varied populations and environments.^30^ Furthermore, using PCA enabled a holistic differentiation of human movement patterns between high- and low-function groups,^15–17^ thereby clarifying which specific aspects of movement are most impacted by LBP. This approach is beneficial in identifying potential biomechanical markers that could inform future therapeutic interventions.

The ADLs assessed in this study were selected to capture common movements that may be impacted by lower back pain. These tasks (such as squat, object pickup, trunk flexion, and sit-to-stand) have been shown to challenge motor control, spinal loading and postural stability, all of which are areas affected by LBP. Previous work has demonstrated that impairments in these movements are associated with reduced quality of life, slower movement speed and altered kinematics among individuals with LBP.^31–35^ As such, these represent ecologically valid challenges that may expose underlying dysfunction in a real-world context. Based on the current results, the movement features derived from the tasks selected were related to both individual PROMs and composite indices of low back function. The data presented here suggest that the movements necessitating spine flexion/extension resulted in the strongest associations with participant reported measures of low back function (both individual and aggregate).

When analyzing discrete variables (movement time and trunk range of motion), our results align with previous research,^36,37^ revealing that individuals with LBP took significantly longer to perform certain ADLs. The whole-body analysis provided deeper insights, revealing clear distinctions between high- and low-function groups. Specifically, high-function individuals consistently exhibited greater joint ranges of motion and faster movements across most ADLs, suggesting more efficient and controlled movement strategies. In contrast, the low-function group frequently showed restricted motion and slower execution. These movement differences may reflect protective or compensatory adaptations, but because task specific pain intensity was not measured during testing, these interpretations remain speculative.

As noted previously, when comparing the composite index models across different movements, the trunk flexion movement presented with the highest correlation coefficient and the cumulative explained variance. Given that trunk flexion demonstrated the strongest association in-sample association with the composite index (R^2^ = 0.71), this movement may be a promising candidate to integrate into a routine clinical workflow, as a brief standardized screening task. A secondary analysis using a more conservative 95% PCA retention threshold was also performed to examine whether the relative task hierarchy was maintained when fewer movement components were retained. Compared with the original 99% PCA threshold, the 95% threshold reduced the number of retained predictors and decreased the magnitude of association across all movement tasks (Table 3). Despite this reduction, trunk flexion remained the strongest movement-specific model across both PCA thresholds, supporting the robustness of its relative association with the composite index. However, the ranked order of association strength changed among the middle-performing tasks. Under the 99% PCA threshold, the order was trunk flexion, small-object pick-up, sit-to-stand, squat and stand-to-sit. Under 95% PCA threshold, the order changed to trunk flexion, sit-to-stand, squat, and stand-to-sit. This shift suggests that the association observed for small-object pick-up in the 99% PCA model may have been more dependent on lower-variance principal components removed by the 95% threshold, whereas sit-to-stand retained a comparatively stronger association under the more conservative PCA threshold. Therefore, while reducing the PCA threshold weakened al models, the primary conclusion that trunk flexion showed the strongest task-level association was maintained.

The current results may suggest utility in objective motor screening in a clinical context. As an example, clinicians could administer a short trunk flexion recording intake in-clinic or remotely to flag patients who may benefit from more detailed assessment or targeted rehabilitation. Because this movement is quick to perform and requires minimal space or equipment it could help with monitoring patients longitudinally. By repeating the same capture at follow-up visits, alongside their PROMs, it could provide an objective measure of change in their movement strategy (e.g. ROM, speed, coordination). Rather than indicating validated predictive performance, these findings suggest that trunk flexion demonstrated the strongest in-sample association with overall low back dysfunction compared with the other movement tasks. Given these insights, tasks requiring trunk flexion could be considered a key focus when developing an automated movement screen aimed at distinguishing low back dysfunction. However, these findings should be interpreted cautiously given the exploratory modelling approach and the need for external validation. At the same time, given the multifaceted nature of low back dysfunction, the inclusion of multiple ADLs provides us with a more holistic assessment, enhancing the quality and accuracy of automated screening tools. Understanding the hierarchy of correlations among different movements (trunk flexion, small object pick-up, sit-to-stand, squat, and stand-to-sit) can help guide the design of an automated movement screen using inputs derived from consumer-grade video. This provides valuable guidance for developing focused clinical assessments that prioritize tasks most strongly correlated with dysfunction.

The integration of PROMs into a composite index allowed for capturing the complexity of the low back dysfunction construct, reflecting the inherently multidimensional nature of LBP as represented in the BPS model.^38,39^ The component PROMs showed expected overlap prior to aggregation, with moderate-to-strong intercorrelations among ODI, QBPDS, PCS, and TSK, while IPAQ showed weak inverse correlations with the other measures. Multicollinearity diagnostics did not indicate severe redundancy, supporting the use of these questionnaires as related but non-identical contributors to the composite index. Among individual PROMs, the QBPDS consistently showed the strongest correlations with movement characteristics, emphasizing the critical role of disability measures in capturing functional impairments related to motor control strategies. Conversely, the IPAQ exhibited the weakest relationships, potentially due to its indirect linkage with specific movement patterns or its susceptibility to self-report biases. Importantly, the composite index constructed with the current study was used as an exploratory summary measure, with future work needed to confirm the robustness and sensitivity of this type of aggregate analysis. Specifically, since the present weighting scheme was selected on conceptual grounds to balance overlapping disability measures against distinct biopsychosocial domains, future work should examine whether alternative weighting strategies produce similar results and should more formally evaluate the relative contribution of psychosocial constructs with comparable composite models. Complimenting the computation of composite indices, aggregate PCA reconstructions across tasks provided clear visual evidence of distinct biomechanical strategies adopted by high- and low-function groups (e.g., Figure 3). Notably, low-function individuals consistently exhibited decreased joint ranges of motion and prolonged movement times, indicative of altered neuromotor strategies to avoid pain exacerbation.^37^ These observations were particularly pronounced in activities that required substantial spinal flexion or dynamic lower-body engagement. Understanding these compensatory patterns can directly inform targeted rehabilitative interventions and real-time clinical assessments.

When closely inspecting aggregate PCA reconstructions, clear temporal differences were evident between the high-, mean-, and low-function groups across all movements. Specifically, the high-function group in the squat, small-object pickup, and sit-to-stand completed their movement tasks first in comparison to the low-function group, while the high-function group in trunk-flexion, completed their task last in comparison to the low-function group. This may be due to different strategies of movement (hip-dominated vs spine-dominated) being used by participants for the trunk flexion movement. Similarly, a study by Lavender et al., (2003) found speed to be a significant contributing factor for loading the spine at the L5/S1 disc during a trunk flexion of a lift. More specifically, this severe trunk flexion has been shown to increase the likelihood of developing low-back disorders.^36^

To specifically quantify joint and segment position/orientation differences across these groups, biomechanical variables (Ankle PF/DF, Knee Flex/Ext, Hip Flex/Ext, Shoulder Flex/Ext, Trunk Inclination, and Pelvis Height) were created (e.g., Figure 3). These are but a subset of the potential outcomes that can be drawn from whole-body point cloud reconstructions. Nevertheless, results demonstrated a relationship between joint range of motion and group classification. It was observed that the low-function group had a lower ROM across all movements, which might be due to individuals with LBP tending to adopt compensatory strategies to minimize their discomfort.^36^ When comparing/contrasting specific movements interesting trends and observations emerge. Specifically, during the small object pick-up, the knee, hip, and shoulders for the low function group displayed a reduced ROM, an increase in trunk inclination angle, and a decrease in pelvis height. This observation is consistent with results typically presented in an LBP population as these individuals may be avoiding bending directly due to back pain and instead squatting completely to avoid stressing their lower back. During the squat movement, a decrease in the ROM for the low-function group is observed for the hip and trunk inclination angle. This might be a result of this group modifying their posture to minimize spinal loading (e.g. less bending of the back, more cautious and slower movement). For the trunk flexion movement, a decrease in trunk inclination angle is observed for the low-function group. Similarly to the other movements, this could be due to the perception of noxious input in flexed spine postures, resulting in less bending of the back. Finally, both sit-to-stand and stand-to-sit movements exhibited similar waveform patterns across function levels, with the primary differences being the reduced amplitude and slower execution of knee and hip motions among low-function participants. These findings are consistent with altered movement behavior in lower-function participants.

A notable methodological strength of this study was its decentralized data collection using widely accessible smartphone technology and open-source pose estimation software (MediaPipe™, Google). Thisapproach significantly increased scalability and accessibility, allowing for broad demographic and geographic representation. Despite this strength, this approach introduced challenges related to participant compliance and consistency. A substantial proportion of videos were excluded due to procedural errors, such as incorrect movement execution, varied camera placements, lighting conditions, and clothing interference with pose estimation. In a real-world clinical workflow, this level of data could reduce utility if recordings are fully self-guided without immediate feedback, by increasing the likelihood of repeat attempts, prolonging assessment time and increasing the likelihood of unusable or lower-confidence outputs. This procedural exclusion may be non-random, affecting individuals with lower digital literacy, mobility limitations, or constrained environments, which could bias results towards participants able to produce higher-quality recordings. Future implementations can mitigate these issues by employing standardized, interactive instructions and automated validation tools, which provide real-time feedback to participants to correct errors (e.g., camera angle or lighting). The system could be implemented as an on-device layer that is continuously monitoring pose landmark visibility/confidence, detect occlusion of key joints (e.g. hips/knees/ankles), verify that the full body remains in frame at an appropriate distance, and flag insufficient illumination or excessive motion blur during the movement. When quality thresholds are not met, the system could prompt actionable corrections (e.g. “step back”, “lower/raise the camera”, “increase the lighting”, or “remove/change clothes obstructing the hips”) and require a brief “quality pass” before upload, thereby reducing procedural exclusions. Moreover, refining pose estimation algorithms through training on diverse datasets (varying clothing, environments, and lighting) could enhance joint-tracking accuracy, further improving reliability.

This study has several limitations. First, the task-specific pain intensity was not measured during testing, so the observed movement patterns cannot be definitively interpreted as pain-avoidance strategies. Second, the reported associations should be interpreted cautiously because the analysis were exploratory. In the primary analysis, a 99% PCA retention threshold was selected to minimize information loss from the high-dimensional whole-body movement data and to preserve subtle movement features that may be relevant to low back dysfunction, including lower-variance components reflecting coordination, timing or task-specific movement strategies. However, this approach also retained a relatively large number of components with the trunk flexion model retaining many predictors (86 PCs + age) relative to the available sample for that task (N = 324), which increases the risk of overfitting. To assess the influence of PCA dimensionality reduction, a secondary analysis using a more conservative 95% PCA retention threshold was performed. This reduced model complexity across all movements and decreased the corresponding R^2^ values, suggesting that the original 99% PCA models likely captured subtle lower-variance movement features that strengthened in-sample associations but also increased model complexity. Nevertheless, trunk flexion remained the strongest movement-specific model under the 95% threshold, indicating that the primary task-level conclusion was maintained when a more conservative PCA approach was used. Importantly, with no cross-validation, bootstrapping, or independent test-set validation being performed, the present models were intended to characterize in-sample associations between movement-derived principal components and low back dysfunction-related PROMs, rather than to establish a validated predictive model. Future work should therefore assess the predictive utility of similar smartphone-based movement analysis approaches using independent validation cohorts, and different model architectures with appropriate cross-validation. In addition, future studies should evaluate the validity and reliability of prediction accuracy across repeated recordings, different environments, and clinically relevant subgroups before these models are implemented as automated screening tools. Multiple task-specific and PROM-specific models were also examined without formal correction for multiple testing. Third, the composite index was used as an exploratory summary measure, and its weighting scheme was selected based on conceptual grounds. Formal psychometric evaluation, such as confirmatory factor analysis or reliability assessment, was not performed in the present study. Finally, although the decentralized smartphone-based dajta collection approach improved scalability and accessibility, it also resulted in substantial video exclusions due to procedural and recording-quality issues, and these exclusions may have been non-random, potentially biasing the sample towards participants that are able to provide higher-quality recordings. Accordingly, these findings should be interpreted as exploratory and should be evaluated in future studies using external validation, improved recording standardization, and more formal evaluation of the composite index.

## 5. Conclusion

This study demonstrates that movement patterns derived from a consumer grade smartphone video are significantly associated with patient-reported measures of low back dysfunction. Across the activities examined, movements involving spinal flexion/extension showed the strongest associations with both individual PROMs and the composite index, suggesting that these tasks may be particularly informative for automated movement-based screening. These findings support the potential of accessible video-based motion analysis as a scalable tool used for assessing functional characteristics related to low back pain. Although an important first step in objective movement analysis, the results should be interpreted as exploratory until confirmed through external validation, improved model generalizability testing, and further evaluation in clinical settings.

## Supplemental material

Supplemental material - Videos of common activities reveal movement patterns associated with biopsychosocial low back function in a worldwide cohort Supplemental material for Videos of common activities reveal movement patterns associated with biopsychosocial low back function in a worldwide cohort by Carl J. Alano, Michael W. R. Holmes, Shawn M. Beaudette in DIGITAL HEALTH

Supplemental material - Videos of common activities reveal movement patterns associated with biopsychosocial low back function in a worldwide cohort Supplemental material for Videos of common activities reveal movement patterns associated with biopsychosocial low back function in a worldwide cohort by Carl J. Alano, Michael W. R. Holmes, Shawn M. Beaudette in DIGITAL HEALTH

## Data Availability

Individual, de-identified participant study data will be shared upon reasonable request for the purpose of replication by contacting the corresponding author SMB.*
