# Supplementary material for: Videos of common activities reveal movement patterns associated with biopsychosocial low back function in a worldwide cohort
Source: Digit Health. 2026 Aug 8;12:20552076261477543. doi: 10.1177/20552076261477543 (PMC13452943; doi:10.1177/20552076261477543)
Supplement: Supplementary file 1 [file sj-pdf-1-dhj-10.1177_20552076261477543.pdf]

SUPPLEMENTARY INFORMATION

Table. Suppl. 1 Intercorrelations and multicollinearity diagnostics among PROMs contributing to the composite index. Values in the matrix are Spearman correlation coefficients (ρ). Pairwise correlations among ODI, QBPDS, PCS, and TSK were all statistically significant at $p < 0.001$. IPAQ showed weak inverse correlations with the other PROMs, and only the ODI–IPAQ correlation reached statistical significance ($p = 0.020$). VIF = variance inflation factor.

Variable	ODI	QBP	PCS	TSK	IPAQ	VIF	Tolerance
**ODI**	1.00	0.77	0.37	0.58	*-0.13*	3.36	0.30
**QBPDS**	0.77	1.00	0.42	0.64	*-0.08*	3.48	0.29
**PCS**	0.37	0.42	1.00	0.49	*-0.06*	1.30	0.77
**TSK**	0.58	0.64	0.49	1.00	*-0.07*	1.78	0.56
**IPAQ**	-0.13	-0.08	-0.06	-0.07	*1.00*	1.02	0.98

Table. Suppl. 2 In-sample model fit statistics for exploratory stepwise linear regression models for the squat task.

	r^2	Adjusted r^2	RMSE	P
Composite Index	0.42	0.33	0.53	<0.001
ODI	0.44	0.36	0.80	<0.001
QBP	0.45	0.37	0.80	<0.001
TSK	0.38	0.30	0.84	<0.001
IPAQ	0.38	0.30	0.84	<0.001
PCS	0.42	0.35	0.81	<0.001

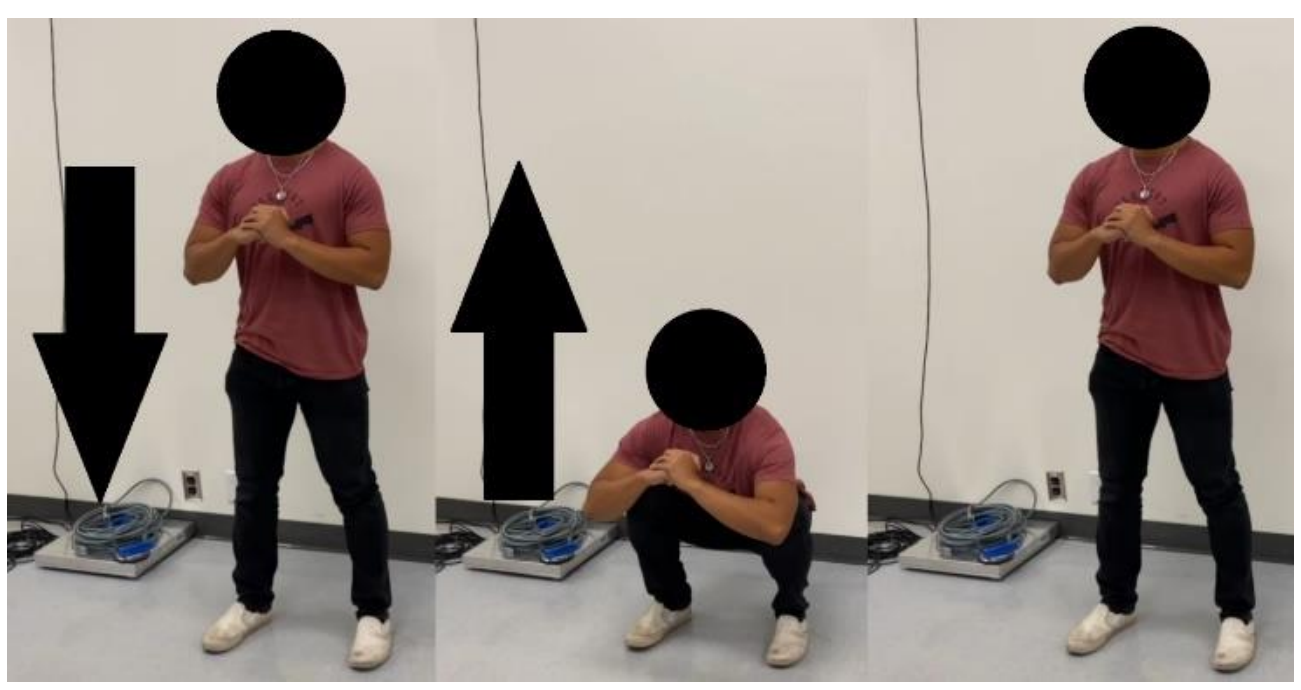

Fig. Suppl. 1. Image demonstrating squat. Participants were required to stand with their legs shoulder width apart, and descend as far as they feel comfortable, at their own self-selected pace, before returning to their original position.

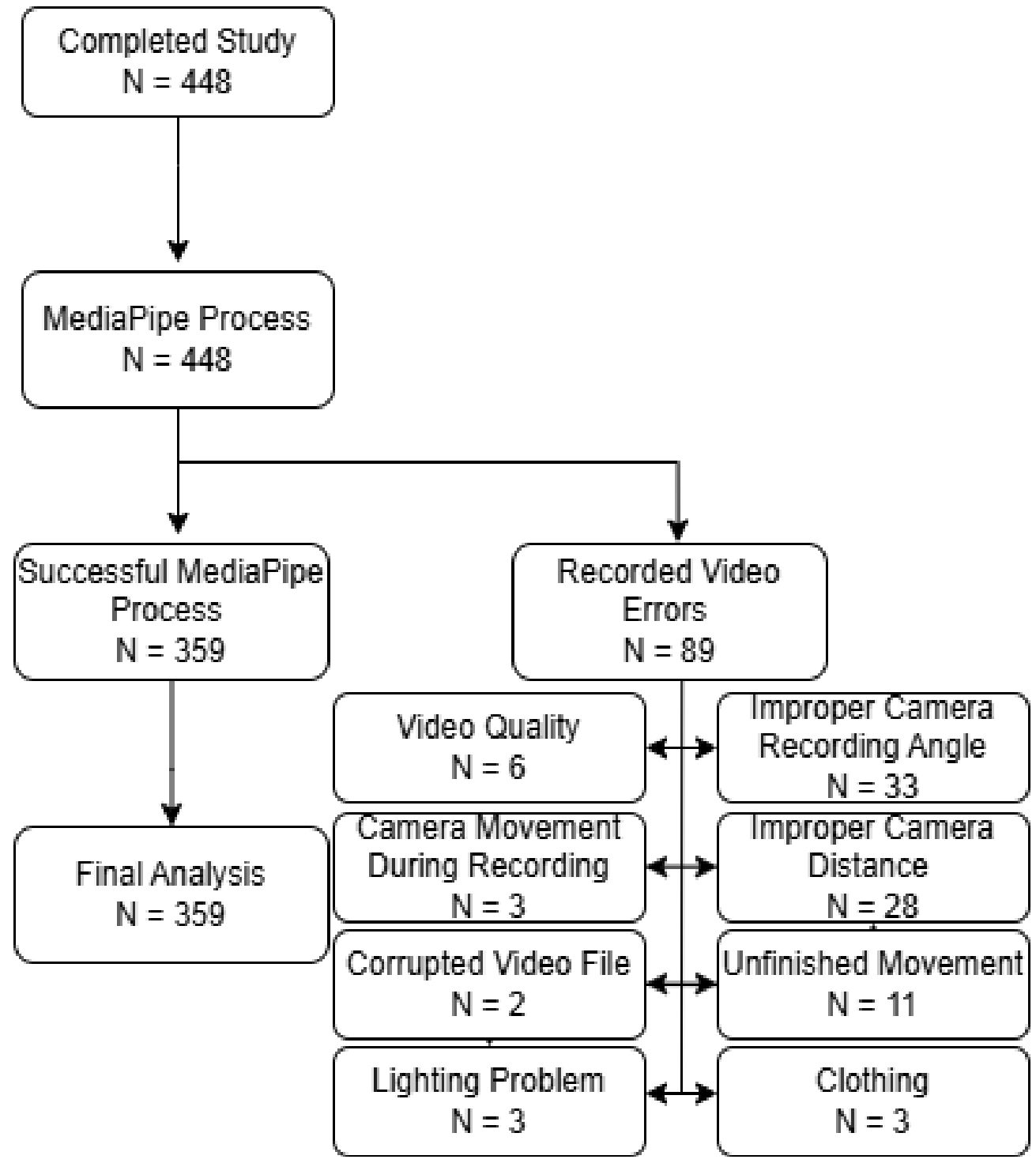

Fig. Suppl. 2. Flowchart of data inclusion/exclusion process for squat movement. The flowcharts detail the steps taken to exclude videos submitted due to errors. It is important to note that these error categories are not mutually exclusive. Some videos might have contained multiple errors; however, each video was only categorized under the error that had the most significant impact on the recording quality.

Table. Suppl. 3 Results of Statistical Analysis for Squat movement

	**Squat (99% pcs retained = 205)**					
**Sample size = 329**	**ODI**	**QBPDS**	**TSK**	**PCS**	**IPAQ**	**Composite index**
**Retained predictors**	41	43	39	36	37	41
**Cumulative explained variance**	15.63	18.36	13.50	13.67	18.52	13.30
**R^2**	0.44	0.45	0.38	0.42	0.38	0.42
**RMSE**	0.80	0.80	0.84	0.81	0.84	0.53
**F stat**	5.61	5.45	4.61	5.95	4.76	5.03
**P-value**	<0.001	<0.001	<0.001	<0.001	<0.001	<0.001
**Kolmogorov-smirnov statistic**	0.11	0.12	0.06	0.09	0.11	0.18

Table. Suppl. 4 In-sample model fit statistics for exploratory stepwise linear regression models for the Lift task.

	r^2	Adjusted r^2	RMSE	P
Composite Index	0.54	0.45	0.48	<0.001
ODI	0.52	0.44	0.75	<0.001
QBP	0.49	0.39	0.78	<0.001
TSK	0.56	0.46	0.73	<0.001
IPAQ	0.35	0.27	0.85	<0.001
PCS	0.45	0.37	0.80	<0.001

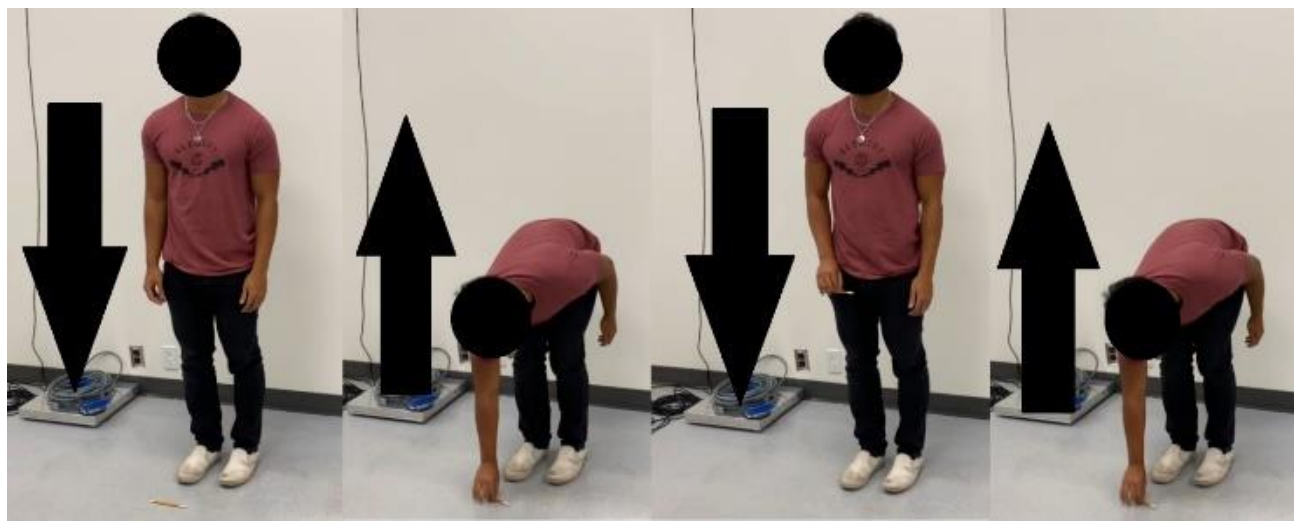

Fig. Suppl. 3. Image demonstrating small-object pickup. Participants were instructed to place the object ~30 cm on the floor directly before their feet. They were then instructed to grasp the object with their right hand and lift the object to their waist height and return to their original position at their own self-selected pace.

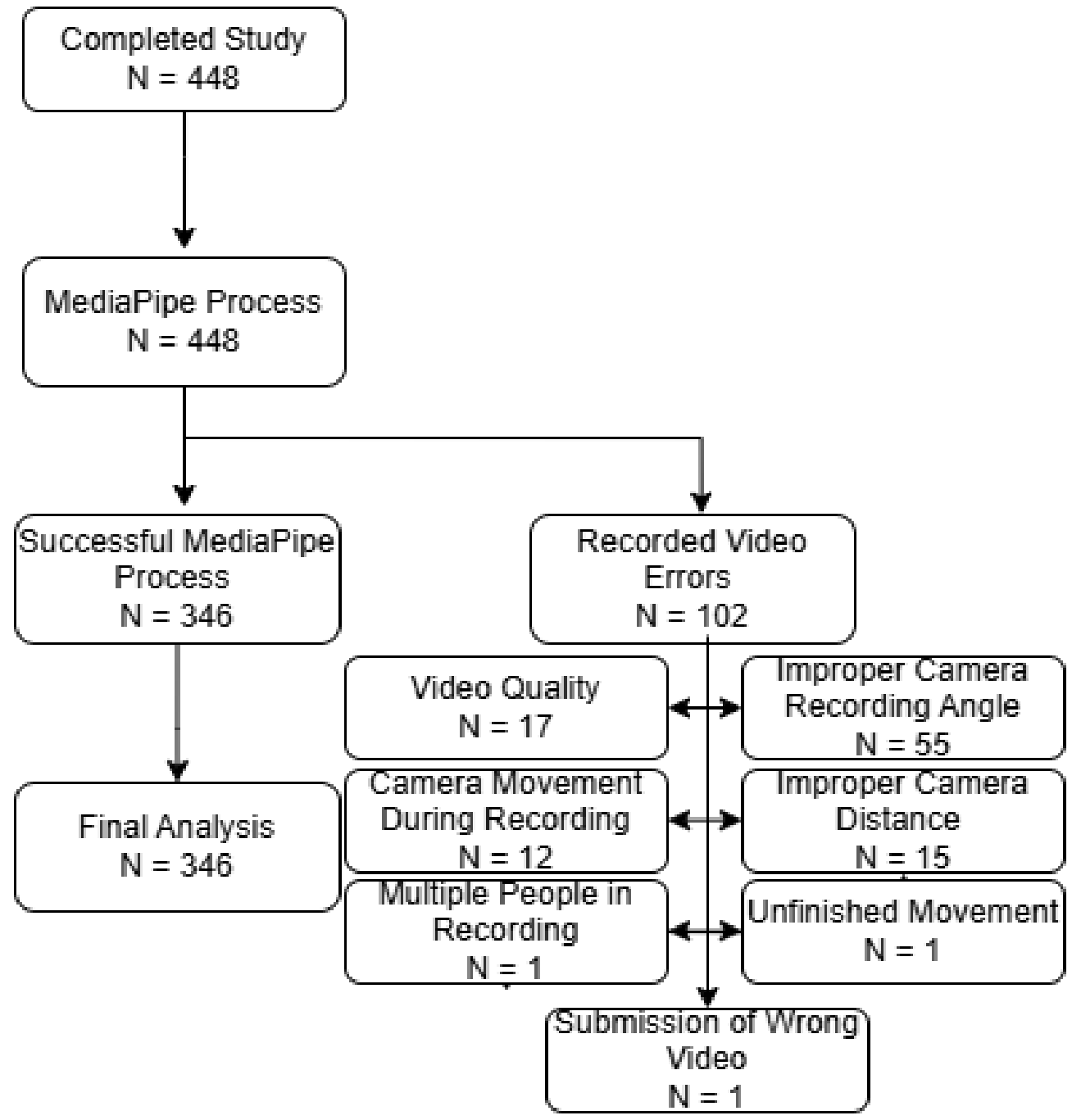

Fig. Suppl. 4. Flowchart of data inclusion/exclusion process for small-object pick-up movement. The flowcharts detail the steps taken to exclude videos submitted due to errors. It is important to note that these error categories are not mutually exclusive. Some videos might have contained multiple errors; however, each video was only categorized under the error that had the most significant impact on the recording quality.

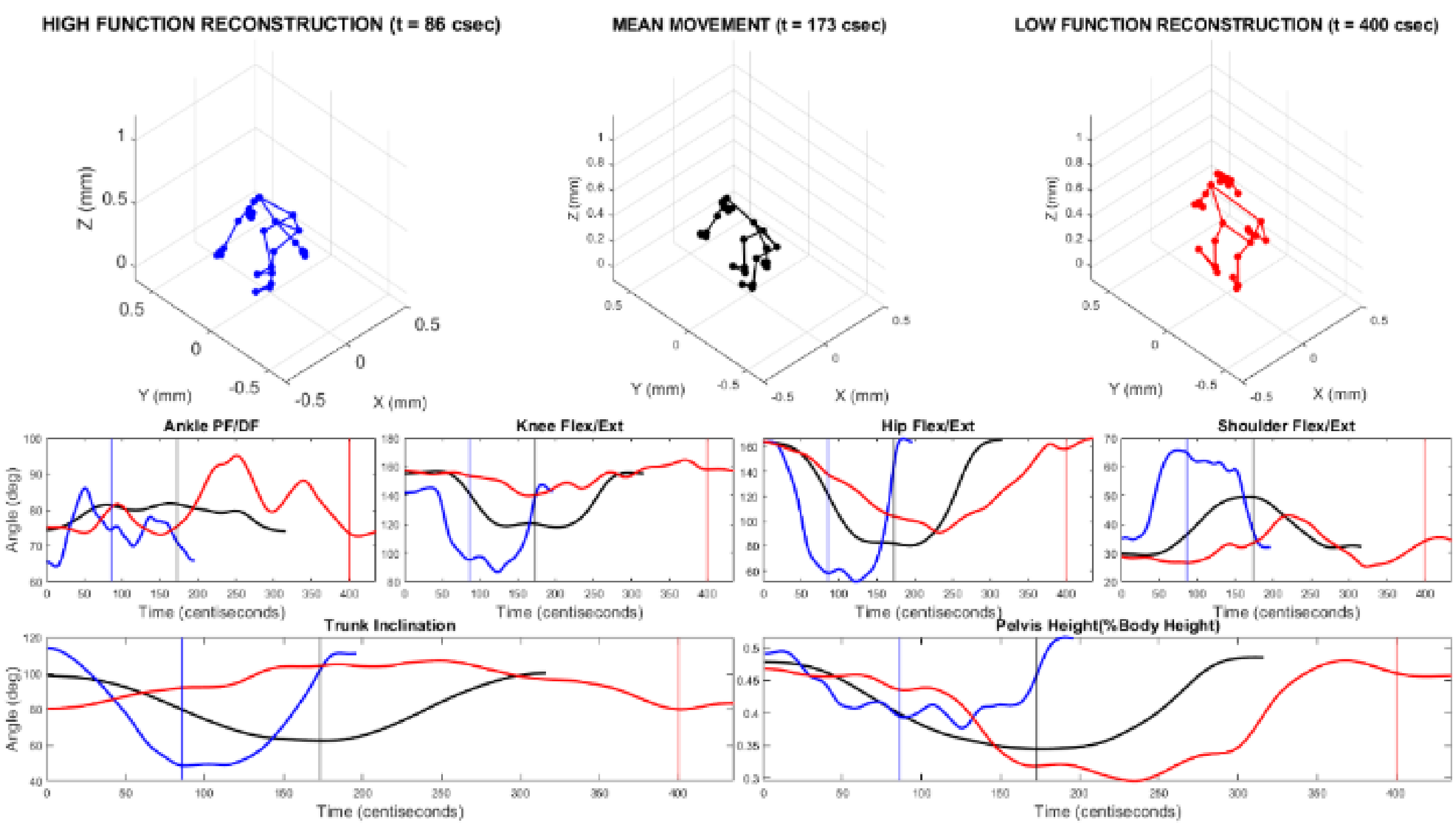

Fig. Suppl. 5. Aggregate reconstruction of small-object pick-up movement at low, mean, and high composite index levels using stepwise linear regression. Movement reconstructions were generated using predicted pca scores from a stepwise linear regression model, with the composite index as the predictor. The reconstructions represent low (5th percentile, red), mean (black), and high (95th percentile, blue) ci values. The top row displays a static 3D pose at the moment of lowest trunk inclination. The bottom row represents kinematic plots showing angular changes over time for various joints and body parts for the entire movement. The vertical lines represent the lowest point of trunk inclination during the movement.

Table. Suppl. 5 Results of Statistical Analysis for Small-object pick-up movement

	Small object pick-up (99% pcs retained = 216)					
**Sample size = 331**	**ODI**	**QBPDS**	**TSK**	**PCS**	**IPAQ**	**Composite index**
**Retained predictors**	48 + age	49 + bmi + age	57 + age	40	33	52 + age
**Cumulative explained variance**	15.05	17.58	23.67	32.19	15.33	25.52
**R^2**	0.52	0.49	0.56	0.45	0.35	0.54
**RMSE**	0.75	0.78	0.73	0.80	0.85	0.48
**F stat**	6.22	5.14	5.88	5.78	4.74	6.03
**P-value**	<0.001	<0.001	<0.001	<0.001	<0.001	<0.001
**Kolmogorov-smirnov statistic**	0.14	0.10	0.12	0.09	0.11	0.23

Table. Suppl. 6 In-sample model fit statistics for exploratory stepwise linear regression models for the Flex task.

	r^2	Adjusted r^2	RMSE	P
Composite Index	0.71	0.61	0.41	<0.001
ODI	0.70	0.59	0.64	<0.001
QBP	0.63	0.52	0.69	<0.001
TSK	0.68	0.59	0.64	<0.001
IPAQ	0.36	0.28	0.85	<0.001
PCS	0.61	0.54	0.68	<0.001

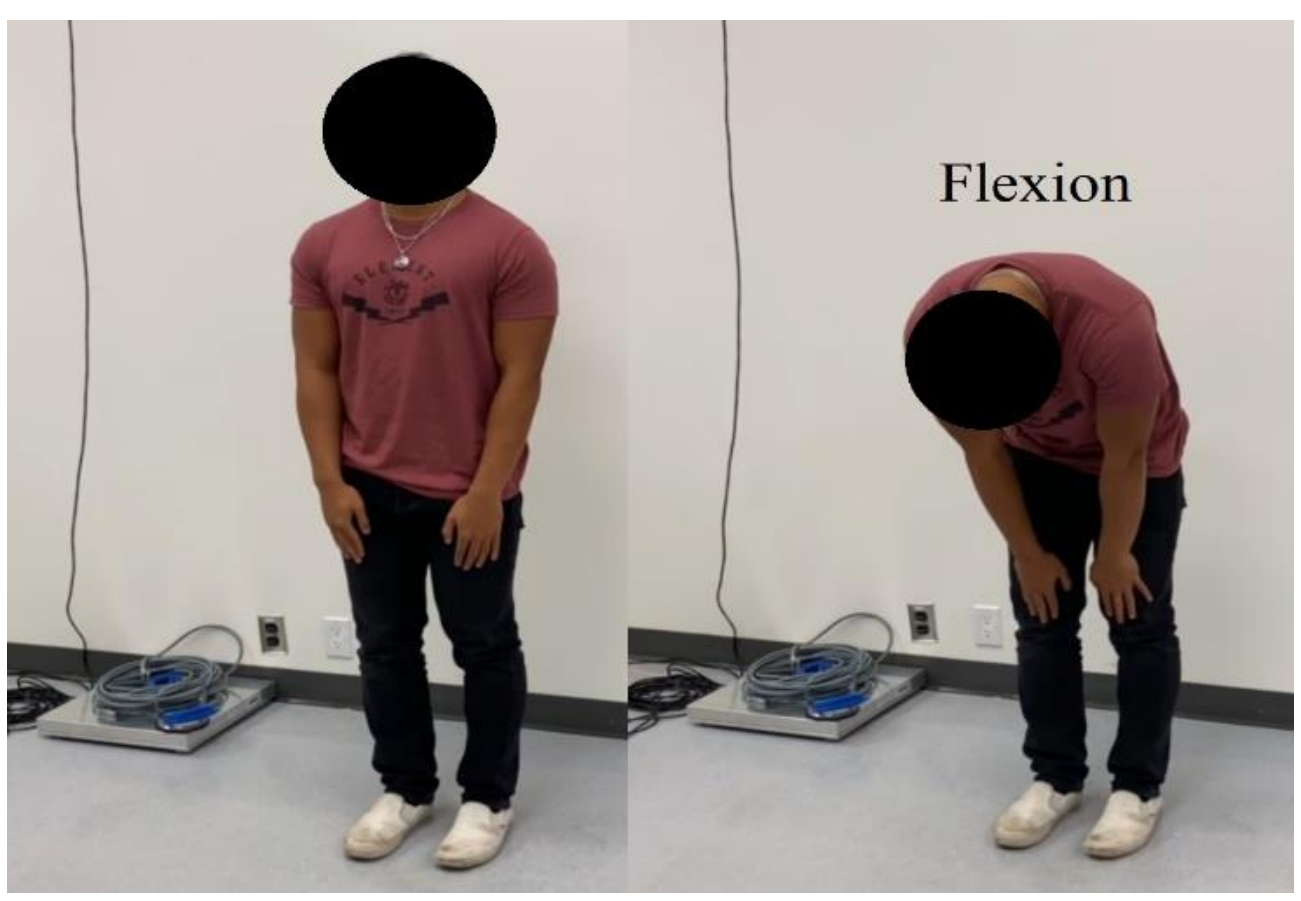

Fig. Suppl. 6. Image demonstrating trunk flexion. Participants were instructed to round their spine as much as they felt comfortable, before returning to their original position.

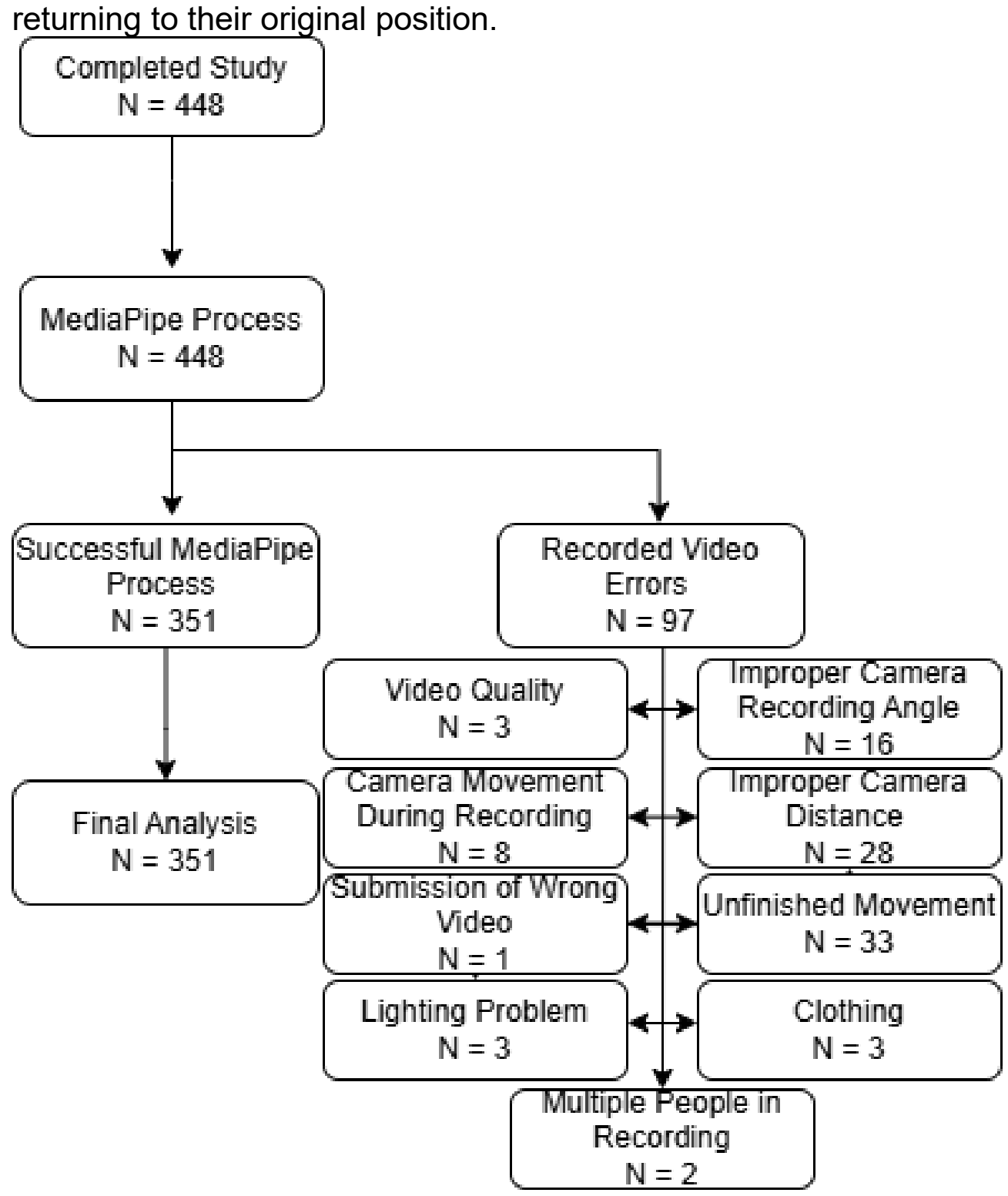

Fig. Suppl. 7. Flowchart of data inclusion/exclusion process for trunk flexion movement. The flowcharts detail the steps taken to exclude videos submitted due to errors. It is important to note that these error categories are not mutually exclusive. Some videos might have contained multiple errors; however, each video was only categorized under the error that had the most significant impact on the recording quality.

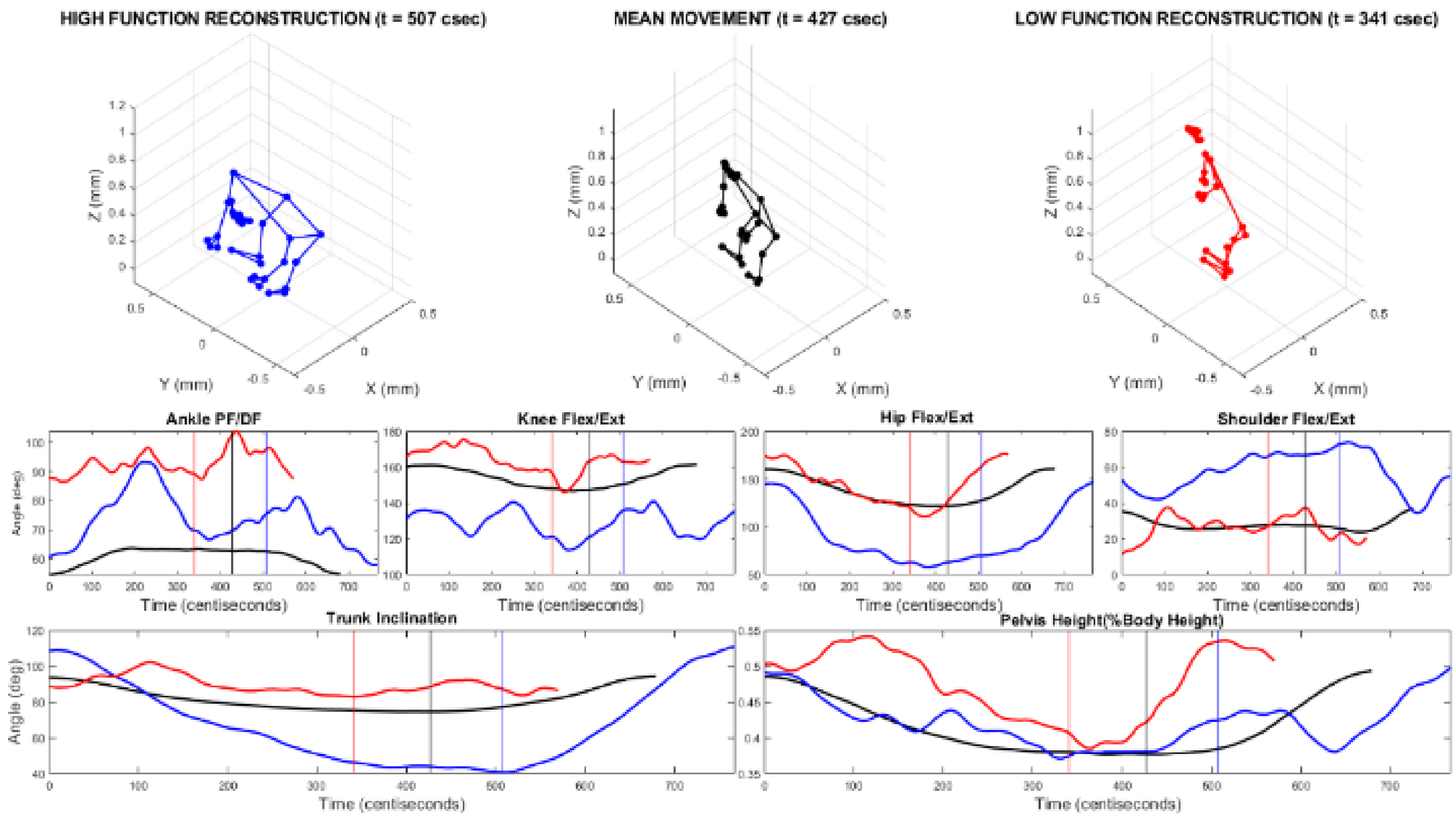

Fig. Suppl. 8. Aggregate reconstruction of trunk flexion movement at low, mean, and high composite index levels using stepwise linear regression. Movement reconstructions were generated using predicted pca scores from a stepwise linear regression model, with the composite index as the predictor. The reconstructions represent low (5th percentile, red), mean (black), and high (95th percentile, blue) ci values. The top row displays a static 3D pose at the moment of lowest trunk inclination. The bottom row represents kinematic plots showing angular changes over time for various joints and body parts for the entire movement. The vertical lines represent the lowest point of trunk inclination during the movement.

Table. Suppl. 7 Results of Statistical Analysis for Trunk Flexion movement

	Trunk flexion (99% pcs retained = 252)					
**Sample size = 324**	**ODI**	**QBPDS**	**TSK**	**PCS**	**IPAQ**	**Composite index**
**Retained predictors**	86 + age	72 + age	72 + bmi + age	74	34	86 + age
**Cumulative explained variance**	36.48	27.48	30.65	31.01	15.26	34.79
**R^2**	0.70	0.63	0.68	0.65	0.36	0.71
**RMSE**	0.64	0.69	0.64	0.68	0.85	0.41
**F stat**	6.30	5.87	7.35	6.22	4.71	6.76
**P-value**	<0.001	<0.001	<0.001	<0.001	<0.001	<0.001
**Kolmogorov-smirnov statistic**	0.18	0.15	0.14	0.15	0.14	0.25

Table. Suppl. 8 In-sample model fit statistics for exploratory stepwise linear regression models for the Sit-2-Stand task.

	r^2	Adjusted r^2	RMSE	P
Composite Index	0.44	0.36	0.52	<0.001
ODI	0.44	0.37	0.80	<0.001
QBP	0.52	0.44	0.75	<0.001
TSK	0.52	0.43	0.76	<0.001
IPAQ	0.41	0.33	0.82	<0.001
PCS	0.42	0.35	0.81	<0.001

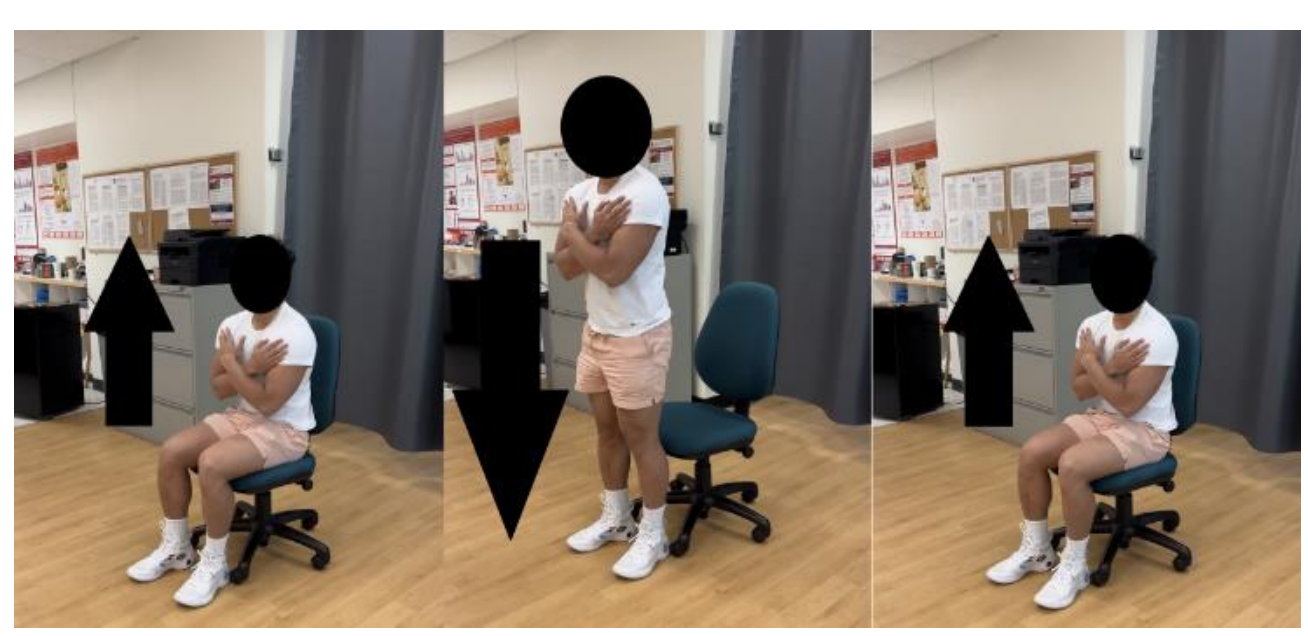

Fig. Suppl. 9. Image demonstrating sit-to-stand and stand-to-sit. Participants were instructed to start seated and have their hands across their chest. While in this position, they were asked to stand and sit back

down, five times at their own self-selected pace. Videos submitted in this movement were split apart into two movements (i.e., sit-to-stand and stand-to-sit) due to the videos not being completed fully.

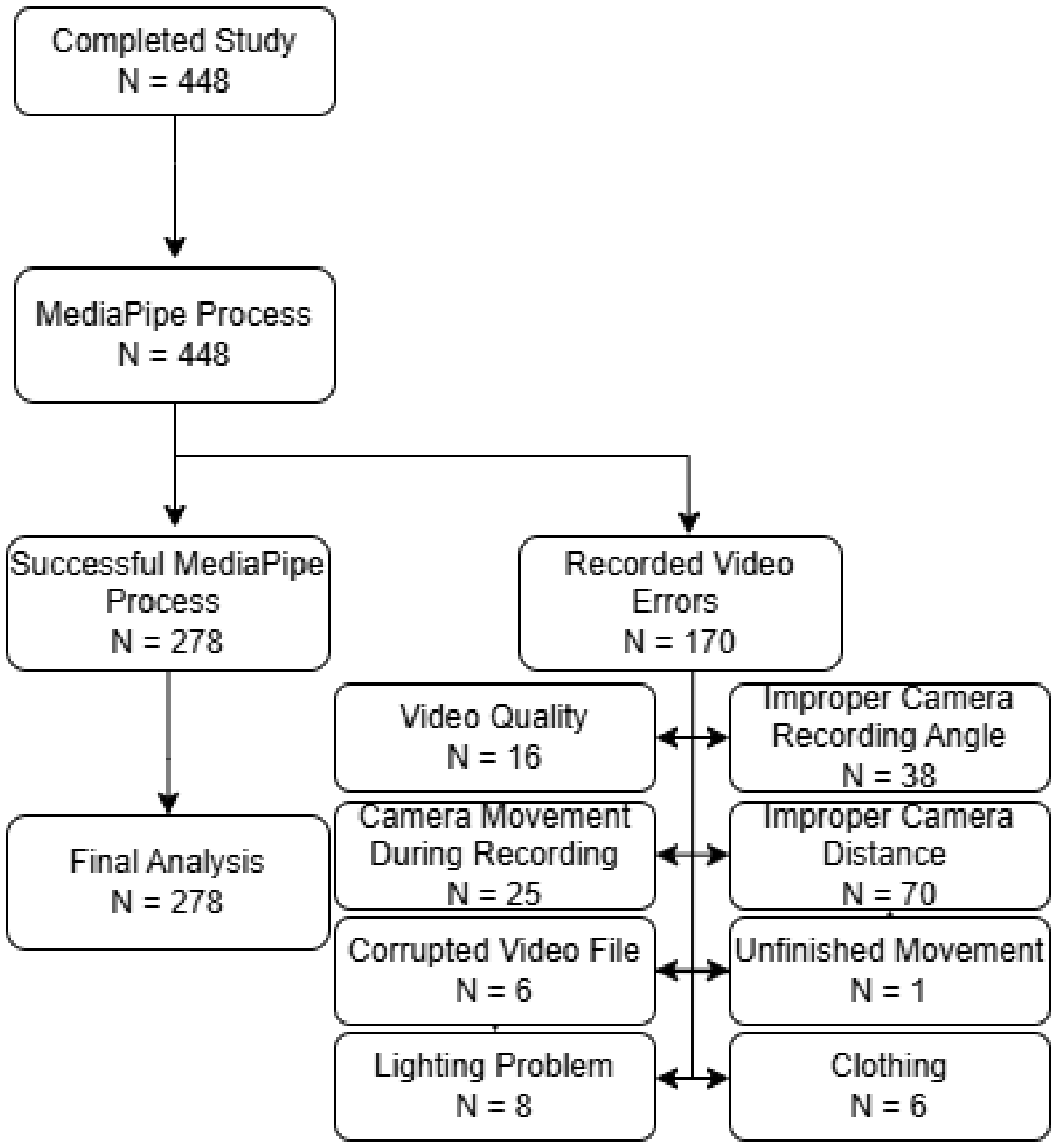

Fig. Suppl. 10. Flowchart of data inclusion/exclusion process for sit-to-stand movement. The flowcharts detail the steps taken to exclude videos submitted due to errors. It is important to note that these error categories are not mutually exclusive. Some videos might have contained multiple errors; however, each video was only categorized under the error that had the most significant impact on the recording quality.

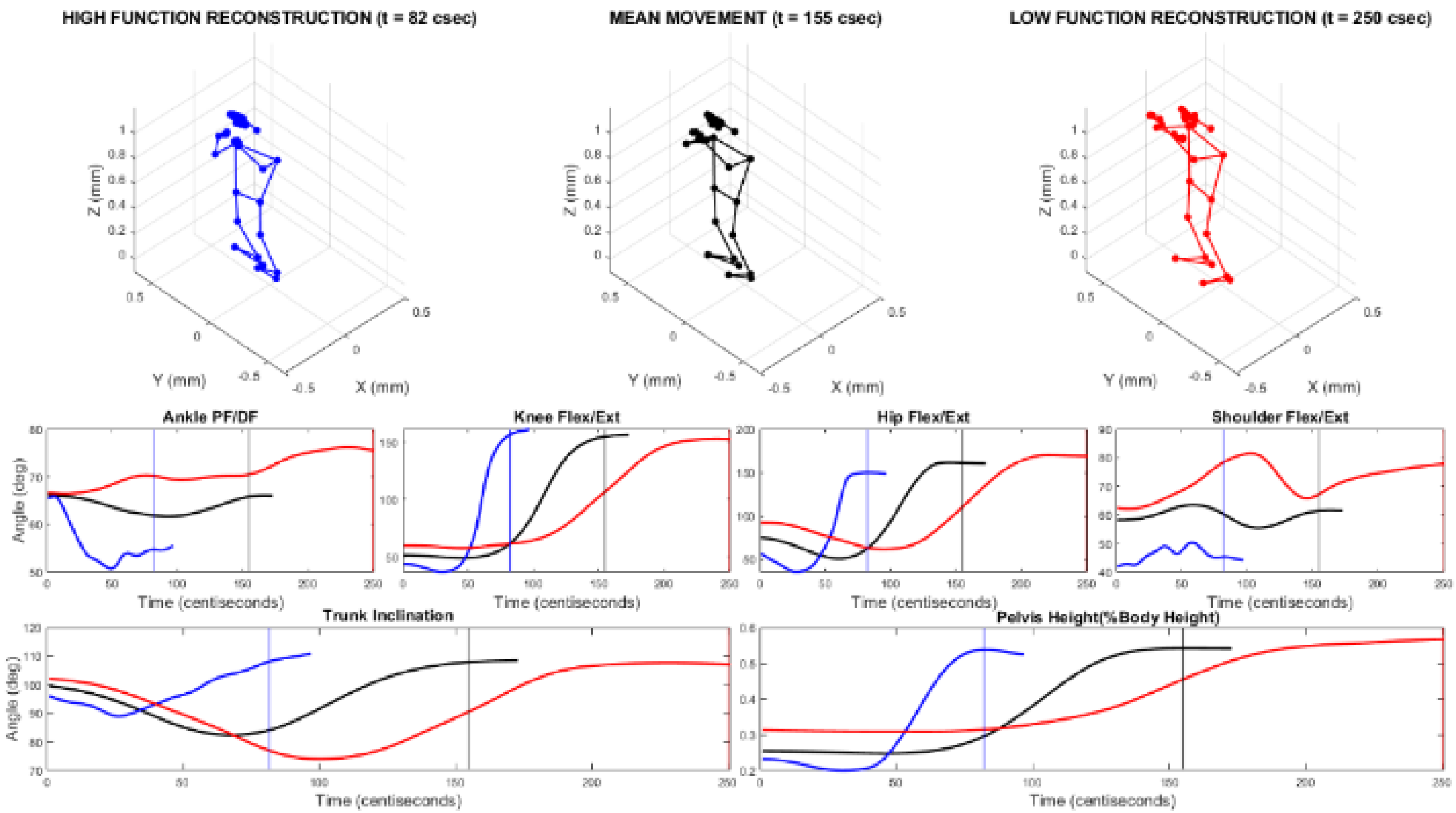

Fig. Suppl. 11. Aggregate reconstruction of sit-to-stand movement at low, mean, and high composite index levels using stepwise linear regression. Movement reconstructions were generated using predicted pca scores from a stepwise linear regression model, with the composite index as the predictor. The reconstructions represent low (5th percentile, red), mean (black), and high (95th percentile, blue) ci values. The top row displays a static 3D pose at the moment of the lowest point of pelvis height. The bottom row represents kinematic plots showing angular changes over time for various joints and body parts for the entire movement. The vertical lines represent the lowest point of pelvis height during the movement.

Table. Suppl. 9 Results of Statistical Analysis for Sit-to-Stand movement

	**Sit-to-stand (99% pcs retained = 171)**					
**Sample size = 262**	**ODI**	**QBPDS**	**TSK**	**PCS**	**IPAQ**	**Composite index**
**Retained predictors**	27 + bmi	34 + bmi + age	38 + bmi	31	32	32 + bmi
**Cumulative explained variance**	17.08	26.02	21.96	22.84	11.78	18.68
**R^2**	0.44	0.52	0.52	0.43	0.41	0.44
**RMSE**	0.80	0.75	0.76	0.81	0.82	0.52
**F stat**	6.47	6.65	6.09	5.47	5.00	5.51
**P-value**	<0.001	<0.001	<0.001	<0.001	<0.001	<0.001
**Kolmogorov-smirnov statistic**	0.12	0.14	0.11	0.10	0.14	0.19

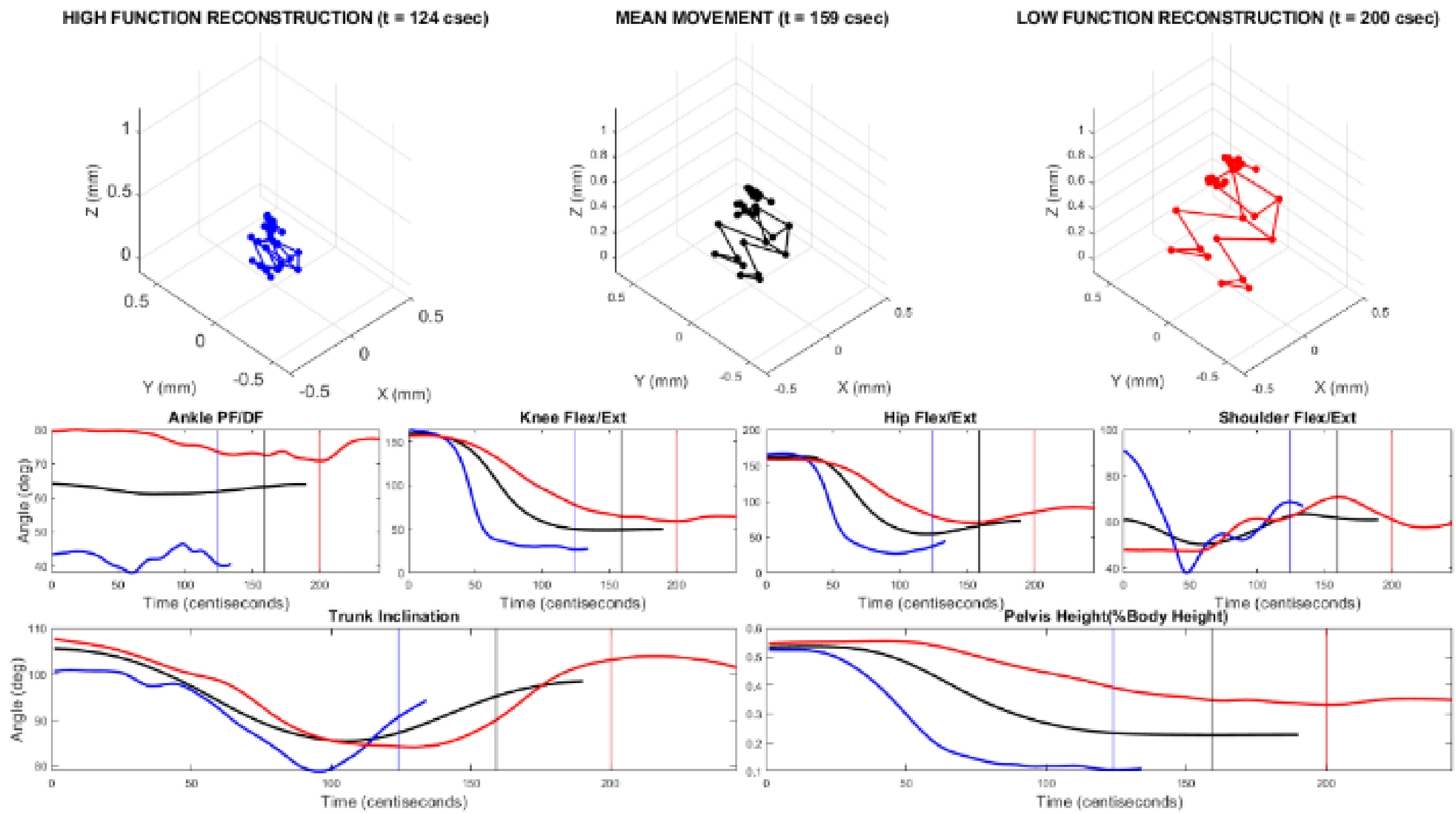

Fig. Suppl. 12. Aggregate reconstruction of stand-to-sit movement at low, mean, and high composite index levels using stepwise linear regression. Movement reconstructions were generated using predicted pca scores from a stepwise linear regression model, with the composite index as the predictor. The reconstructions represent low (5th percentile, red), mean (black), and high (95th percentile, blue) ci values. The top row displays a static 3D pose at the moment of the lowest point of pelvis height. The bottom row represents kinematic plots showing angular changes over time for various joints and body parts for the entire movement. The vertical lines represent the lowest point of pelvis height during the movement.

Fig. Suppl. 13. Flowchart of data inclusion/exclusion process for stand-to-sit movement. The flowcharts detail the steps taken to exclude videos submitted due to errors. It is important to note that these error categories are not mutually exclusive. Some videos might have contained multiple errors; however, each video was only categorized under the error that had the most significant impact on the recording quality.

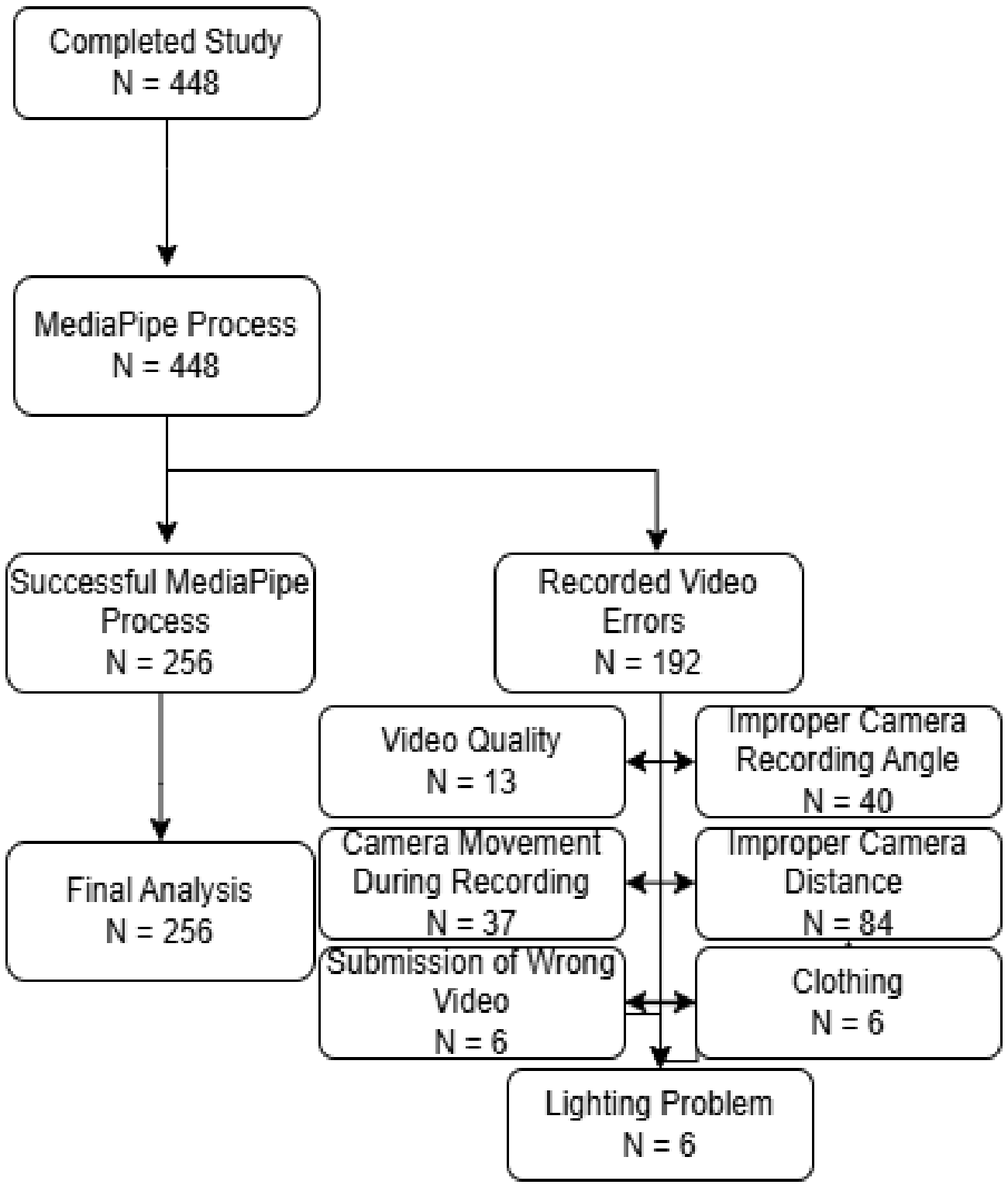

Table. Suppl. 10 In-sample model fit statistics for exploratory stepwise linear regression models for the Stand-2-Sit task.

	r^2	Adjusted r^2	RMSE	P
Composite Index	0.41	0.33	0.53	<0.001
ODI	0.38	0.31	0.83	<0.001
QBP	0.50	0.43	0.76	<0.001
TSK	0.45	0.37	0.80	<0.001
IPAQ	0.29	0.23	0.88	<0.001
PCS	0.34	0.28	0.85	<0.001

Table. Suppl. 11 Results of Statistical Analysis for Stand-to-Sit movement

	**Stand-to-sit (99% pcs retained = 162)**					
**Sample size = 239**	**ODI**	**QBP DS**	**TSK**	**PCS**	**IPAQ**	**Composite index**
**Retained predictors**	23 + bmi	28 + bmi + age	30 + bmi+ age	22	18 + age	28 + bmi + age
**Cumulative explained variance**	4.95	15.58	16.51	6.09	4.35	10.99
**R^2**	0.38	0.50	0.45	0.35	0.29	0.41
**RMSE**	0.83	0.76	0.80	0.85	0.88	0.53
**F stat**	5.50	6.97	5.29	5.16	4.66	4.86
**P-value**	<0.001	<0.001	<0.001	<0.001	<0.001	<0.001
**Kolmogorov-smirnov statistic**	0.10	0.12	0.12	0.09	0.12	<0.001
