# Supplementary material for: Videos of common activities reveal movement patterns associated with biopsychosocial low back function in a worldwide cohort
Source: Digit Health. 2026 Aug 8;12:20552076261477543. doi: 10.1177/20552076261477543 (PMC13452943; doi:10.1177/20552076261477543)
Supplement: Supplementary file 2 [file sj-pdf-2-dhj-10.1177_20552076261477543.pdf]

Questionnaires:

<u>Informed Consent:</u>
I agree to participate in the study described above. I have made this decision based on the information I have read in the Informed-Consent Letter. I understand that I may withdraw with this consent at any time.

YES/NO

Does your age fall within the range of 18-85 years?

YES/NO

Can you read and comprehend the English Language?

YES/NO

Do you have access to a web enabled device, with a digital camera?

YES/NO

Are you healthy and free of any neuromusculoskeletal disorders or ailments (unrelated to the low back/spine)?

YES/NO

Have you ever been classified as having a musculoskeletal (e.g. Parkinson's Disease or Cerebral Palsy) or Neurological (e.g. Diabetic Neuropathy) or auditory (e.g., hearing disorder, etc.) disorder which may affect your **<u>movement or balance</u>**?

YES/NO

Have you ever experienced an injury, for which you sought medical treatment, to your **<u>lower limb</u>** (e.g ankle, knee or hip) within the last three months?

YES/NO

Have you ever experienced pain in the **<u>low back region</u>** that has caused you to miss school, work or any regular activity?

YES/NO (If Yes, please describe)

Have you ever sought medical treatment (physician, chiropractor, physiotherapist) relating to your **<u>low back region</u>**?

YES/NO (If Yes, please describe)

Do you have another individual available to facilitate the recording of your movements using a web-enabled device with a digital camera?

YES/NO

Age:

Sex:

- Male
- Female
- Other

Weight (lbs):

Height (ft):

Which race or ethnicity best describes you? (Please choose only one.)

- White (Eg: German, Irish, English, Italian, Polish, French, etc)
- Hispanic, Latino or Spanish origin (Eg: Mexican or Mexican American, Puerto Rican, Cuban, Salvadoran, Dominican, Colombian, etc)
- Black or African American (Eg: African American, Jamaican, Haitian, Nigerian, Ethiopian, Somalian, etc)
- Asian (Eg: Chinese, Filipino, Asian Indian, Vietnamese, Korean, Japanese, etc)
- American Indian or Alaska Native(Eg: Navajo nation, Blackfeet tribe, Mayan, Aztec, Native Village or Barrow Inupiat Traditional Government, Nome Eskimo Community, etc)
- Middle Eastern or North African (Eg: Lebanese, Iranian, Egyptian, Syrian, Moroccan, Algerian, etc)
- Native Hawaiian or Other Pacific Islander (Eg: Native Hawaiian, Samoan, Chamorro, Tongan, Fijian, etc)
- Some other race, ethnicity or origin (Please specify)

- White (Eg: German, Irish, English, Italian, Polish, French, etc)
- Hispanic, Latino or Spanish origin (Eg: Mexican or Mexican American, Puerto Rican, Cuban, Salvadoran, Dominican, Colombian, etc)
- Black or African American (Eg: African American, Jamaican, Haitian, Nigerian, Ethiopian, Somalian, etc)
- Asian (Eg: Chinese, Filipino, Asian Indian, Vietnamese, Korean, Japanese, etc)
- American Indian or Alaska Native(Eg: Navajo nation, Blackfeet tribe, Mayan, Aztec, Native Village or Barrow Inupiat Traditional Government, Nome Eskimo Community, etc)
- Middle Eastern or North African (Eg: Lebanese, Iranian, Egyptian, Syrian, Moroccan, Algerian, etc)
- Native Hawaiian or Other Pacific Islander (Eg: Native Hawaiian, Samoan, Chamorro, Tongan, Fijian, etc)
- Some other race, ethnicity or origin (Please specify)

OSWESTRY LOW BACK DISABILITY QUESTIONNAIRE

Instructions: this questionnaire has been designed to give us information as to how your back pain has affected your ability to manage everyday life. Please answer every section and mark in each section only the ONE box which applies to you at this time. We realize you may consider 2 of the statements in any section may relate to you, but please mark the box which most closely describes your current condition.

1. PAIN INTENSITY

- I can tolerate the pain I have without having to use pain killers
- The pain is bad but I manage without taking pain killers
- Pain killers give complete relief from pain
- Pain killers give moderate relief from pain
- Pain killers give very little relief from pain
- Pain killers have no effect on the pain and I do not use them

2. PERSONAL CARE (e.g. Washing, Dressing)

- I can look after myself normally without causing extra pain
- I can look after myself normally but it causes extra pain
- It is painful to look after myself and I am slow and careful
- I need some help but manage most of my personal care
- I need help every day in most aspects of self care

3. LIFTING

- I can lift heavy weights without extra pain
- I can lift heavy weights but it gives extra pain
- Pain prevents me from lifting heavy weights off the floor, but I can manage if they are conveniently positioned, i.e. on a table
- Pain prevents me from lifting heavy weights, but I can manage light to medium weights if they are conveniently positioned
- I can lift very light weights
- I cannot lift or carry anything at all

4. WALKING

- Pain does not prevent me walking any distance
- Pain prevents me walking more than one mile
- Pain prevents me walking more than ½ mile
- Pain prevents me walking more than ¼ mile
- I can only walk using a stick or crutches
- I am in bed most of the time and have to crawl to the toilet

5. SITTING

- I can sit in any chair as long as I like
- I can only sit in my favorite chair as long as I like
- Pain prevents me from sitting more than one hour
- Pain prevents me from sitting more than ½ hour
- Pain prevents me from sitting more than 10 minutes
- Pain prevents me from sitting at all

6. STANDING

- I can stand as long as I want without extra pain
- I can stand as long as I want but it gives me extra pain
- Pain prevents me from standing for more than one hour
- Pain prevents me from standing for more than 30 minutes
- Pain prevents me from standing for more than 10 minutes
- Pain prevents me from standing at all

7. SLEEPING

- Pain does not prevent me from sleeping well
- I can sleep well only by using medication
- Even when I take medication, I have less than 6 hrs sleep
- Even when I take medication, I have less than 4 hrs sleep
- Even when I take medication, I have less than 2 hrs sleep
- Pain prevents me from sleeping at all

8. SOCIAL LIFE

- My social life is normal and gives me no extra pain
- My social life is normal but increases the degree of pain
- Pain has no significant effect on my social life apart from limiting my more energetic interests, i.e. dancing, etc.
- Pain has restricted my social life and I do not go out as often
- Pain has restricted my social life to my home
- I have no social life because of pain

9. TRAVELLING

I can travel anywhere without extra pain

I can travel anywhere but it gives me extra pain

Pain is bad, but I manage journeys over 2 hours

Pain restricts me to journeys of less than 1 hour

Pain restricts me to short necessary journeys under 30 minutes

Pain prevents me from traveling except to the doctor or hospital

10. EMPLOYMENT / HOMEMAKING

My normal homemaking/ job activities do not cause pain.

My normal homemaking/ job activities increase my pain, but I can still perform all that is required of me.

I can perform most of my homemaking/ job duties, but pain prevents me from performing more physically stressful activities (e.g. lifting, vacuuming)

Pain prevents me from doing anything but light duties.

Pain prevents me from doing even light duties.

Pain prevents me from performing any job or homemaking chores.

The Quebec Back Pain Disability Scale:

This questionnaire is about the way your back pain is affecting your daily life. People with back problems may find it difficult to perform some of their daily activities. We would like to know if you find it difficult to perform any of the activities listed below, because of your back. For each activity there is a scale of 0 to 5. Please choose one response option for each activity (do not skip any activities) and write out the corresponding number.

Today, do you find it difficult to perform the following activities because of your back?

1. Get out of bed

- Not difficult at all
- Minimally difficult
- Somewhat difficult
- Fairly difficult
- Very difficult
- Unable to do

2. Sleep through the night

- Not difficult at all
- Minimally difficult
- Somewhat difficult
- Fairly difficult
- Very difficult
- Unable to do

3. Turn over in bed

- Not difficult at all
- Minimally difficult
- Somewhat difficult
- Fairly difficult
- Very difficult
- Unable to do

4. Ride in a car

- Not difficult at all
- Minimally difficult
- Somewhat difficult
- Fairly difficult
- Very difficult
- Unable to do

5. Stand up for 20-30 minutes

- Not difficult at all
- Minimally difficult
- Somewhat difficult
- Fairly difficult
- Very difficult
- Unable to do

6. Sit in a chair for several hours

- Not difficult at all
- Minimally difficult
- Somewhat difficult
- Fairly difficult
- Very difficult
- Unable to do

7. Climb one flight of stairs

- Not difficult at all
- Minimally difficult
- Somewhat difficult
- Fairly difficult
- Very difficult
- Unable to do

8. Walk a few blocks (300 - 400 m)

- Not difficult at all
- Minimally difficult
- Somewhat difficult
- Fairly difficult
- Very difficult
- Unable to do

9. Walk several kilometres

- Not difficult at all
- Minimally difficult
- Somewhat difficult
- Fairly difficult
- Very difficult
- Unable to do

10. Reach up to high shelves

- Not difficult at all
- Minimally difficult
- Somewhat difficult
- Fairly difficult
- Very difficult
- Unable to do

11. Throw a ball

- Not difficult at all
- Minimally difficult
- Somewhat difficult
- Fairly difficult
- Very difficult
- Unable to do

12. Run one block (about 100m)

- Not difficult at all
- Minimally difficult
- Somewhat difficult
- Fairly difficult
- Very difficult
- Unable to do

13. Take food out of the refrigerator

- Not difficult at all
- Minimally difficult
- Somewhat difficult
- Fairly difficult
- Very difficult
- Unable to do

14. Make your bed

- Not difficult at all
- Minimally difficult
- Somewhat difficult
- Fairly difficult
- Very difficult
- Unable to do

15. Put on socks (pantyhose)

- Not difficult at all
- Minimally difficult
- Somewhat difficult
- Fairly difficult
- Very difficult
- Unable to do

16. Bend over to clean the bathtub

- Not difficult at all
- Minimally difficult
- Somewhat difficult
- Fairly difficult
- Very difficult
- Unable to do

17. Move a chair

- Not difficult at all
- Minimally difficult
- Somewhat difficult
- Fairly difficult
- Very difficult
- Unable to do

18. Pull or push heavy doors

- Not difficult at all
- Minimally difficult
- Somewhat difficult
- Fairly difficult
- Very difficult
- Unable to do

19. Carry two bags of groceries

- Not difficult at all
- Minimally difficult
- Somewhat difficult
- Fairly difficult
- Very difficult
- Unable to do

20. Lift and carry a heavy suitcase

- Not difficult at all
- Minimally difficult
- Somewhat difficult
- Fairly difficult
- Very difficult
- Unable to do

TAMPA SCALE FOR KINESIOPHOBIA (TSK)

Instructions: Please answer the following questions according to your true feelings, not according to what others think you should believe. Score each statement from strongly disagree to strong agree by tapping the appropriate box.

	Strongly disagree	Somewhat disagree	Somewhat agree	Strongly agree
I'm afraid that I might injury myself if I exercise	O	O	O	O
If I were to try to overcome it, my pain would increase	O	O	O	O
My body is telling me I have something dangerously wrong	O	O	O	O
My pain would probably be relieved if I were to exercise	O	O	O	O
People aren't taking my medical condition seriously enough	O	O	O	O
My accident has put my body at risk for the rest of my life	O	O	O	O
Pain always means I have injured my body	O	O	O	O
Just because something aggravates my pain does not mean it is dangerous	O	O	O	O
I am afraid that I might injure myself accidentally	O	O	O	O
Simply being careful that I do not make any unnecessary movements is the safest thing I can do to prevent my pain from worsening	O	O	O	O
I wouldn't have this much pain if there weren't something potentially dangerous going on in my body	O	O	O	O
Although my condition is painful, I would be better off if I were physically active	O	O	O	O
Pain lets me know when to stop exercising so that I don't injure	O	O	O	O
It's really not safe for a person with a condition like mine to be physically active	O	O	O	O
I can't do all the things normal people do because it's too easy for me to get injured	O	O	O	O
Even though something is causing me a lot of pain, I don't think it's actually dangerous	O	O	O	O
No one should have to exercise when he/she is in pain	O	O	O	O

We are interested in finding out about the kinds of physical activities that people do as part of their everyday lives. The questions will ask you about the time you spent being physically active in the last 7 days. Please answer each question even if you do not consider yourself to be an active person. Please think about the activities you do at work, as part of your house and yard work, to get from place to place, and in your spare time for recreation, exercise or sport.

Think about all the vigorous and moderate activities that you did in the last 7 days. Vigorous physical activities refer to activities that take hard physical effort and make you breathe much harder than normal. Moderate activities refer to activities that take moderate physical effort and make you breathe somewhat harder than normal.

Part 1: Job Related Physical Activity

The first section is about your work. This includes paid jobs, farming, volunteer work, course work, and any other unpaid work that you did outside your home. Do not include unpaid work you might do around your home, like housework, yard work, general maintenance, and caring for your family. These are asked in Part 3.

1. Do you currently have a job or do any unpaid work outside your home?

Yes

No

The next questions are about all the physical activity you did in the **last 7 days** as part of your paid or unpaid work. This does not include travelling to and from work.

2. During the **last 7 days**, on how many days did you do **vigorous** physical activities like heavy lifting, digging, heavy construction, or climbing up stairs **as part of your work**? Think about only those physical activities that you did for at least 10 minutes at a time.

days per week

No vigorous job-related physical activity

3. How much time did you usually spend on one of those days doing **vigorous** physical activities as part of your work?

Hours per day

Minutes per day

4. Again, think about only those physical activities that you did for at least 10 minutes at a time. During the **last 7 days**, on how many days did you do **moderate** physical activities like carrying light loads **as part of your work?** Please do not include walking.

days per week

No moderate job-related physical activity

5. How much time did you usually spend on one of those days doing **moderate** physical activities as part of your work?

Hours per day ☐
Minutes per day ☐

6. During the **last 7 days**, on how many days did you **walk** for at least 10 minutes at a time **as part of your work?** Please do not count any walking you did to travel to or from work.

days per week ☐

No job-related walking

7. How much time did you usually spend on one of those days **walking** as part of your work?

hours per day ☐
minutes per day ☐

Part 2: Transportation Physical Activity

These questions are about how you travelled from place to place, including to places like work, stores, movies, and so on.

8. During the **last 7 days**, on how many days did you **travel in a motor vehicle** like a train, bus, car or tram?

days per week ☐

No travelling in a motor vehicle

9. How much time did you usually spend on one of those days travelling in a train, bus, car, tram, or other kinds of motor vehicle?

Hours per day ☐
Minutes per day ☐

10. During the **last 7 days**, on how many days did you use a bicycle for at least 10 minutes at a time to go **from place to place**?

days per week ☐

No bicycling from place to place

11. How much time did you usually spend on one of those days riding a **bicycle** from place to place?

hours per day ☐
minutes per day ☐

12. During the **last 7 days**, on how many days did you **walk** for at least 10 minutes at a time to go **from place to place?**

days per week

No walking from place to place

13. How much time did you usually spend on one of those days walking from place to place?

hours per day

minutes per day

Part 3: Housework, House maintenance, and Caring for Family

This section is about some of the physical activities you might have done in the **last 7 days** in and around your home, like housework, gardening, yard work, general maintenance work, and caring for your family.

14. Think about only those physical activities that you did for at least 10 minutes at a time. During the **last 7 days**, on how many days did you do **vigorous** physical activities like heavy lifting, chopping wood, shoveling snow, or digging **in the garden or yard?**

days per week

No vigorous activity in garden or yard

15. How much time did you usually spend on one of those days doing **vigorous** physical activities in the garden or yard?

hours per day

minutes per day

16. Again, think about only those physical activities that you did for at least 10 minutes at a time. During the **last 7 days**, on how many days did you do **moderate** activities like carrying light loads, sweeping, washing windows, and raking **in the garden or yard?**

days per week

No moderate activity in garden or yard

17. How much time did you usually spend on one of those days doing **moderate** physical activities in the garden or yard?

hours per day

minutes per day

18. Once again, think about only those physical activities that you did for at least 10 minutes at a time. During the **last 7 days**, on how many days did you do **moderate** activities like carrying light loads, washing windows, scrubbing floors and sweeping **inside your home?**

days per week

No moderate activity inside home

19. How much time did you usually spend on one of those days doing **moderate** physical activities inside your home?

hours per day

minutes per day

Part 4: Recreation, Sport and Leisure-time Physical Activity

This section is about all the physical activities that you did in the **last 7 days** solely for recreation, sport, exercise or leisure. Please do not include any activities you have already mentioned.

20. Not counting any walking you have already mentioned, during the **last 7 days,** on how many days did you **walk** for at least 10 minutes at a time **in your leisure time?**

days per week

No walking leisure time

21. How much time did you usually spend on one of those days **walking** in your leisure time?

Hours per days

Minutes per day

22. Think about only those physical activities that you did for at least 10 minutes at a time. During the **last 7 days**, on how many days did you do **vigorous** physical activities like aerobics, running, fast bicycling, or fast swimming **in your leisure time?**

days per week

No vigorous activity in leisure time

23. How much time did you usually spend on one of those days doing **vigorous** physical activities in your leisure time?

hours per day

minutes per day

24. Again, think about only those physical activities that you did for at least 10 minutes at a time. During the **last 7 days**, on how many days did you do **moderate** physical activities like bicycling at a regular pace, swimming at a regular pace, and doubles tennis, etc, **in your leisure time?**

days per week

No moderate activity in leisure time

25. How much time did you usually spend on one of those days doing moderate physical activities in your leisure time?

hours per day
minutes per day

Part 5: Time Spend Sitting

The last questions are about the time you spend sitting while at work, at home, while doing course work and during leisure time. This may include time spent sitting at a desk, visiting friends, reading or sitting or lying down to watch television. Do not include any time spent sitting in a motor vehicle that you already told me about.

26. During the **last 7 days**, how much time did you usually spend **sitting** on a **weekday**?

hours per day
minutes per day

27. During the **last 7 days**, how much time did you usually spend **sitting** on a **weekend day**?

hours per day
minutes per day
